# A new activity model for biotite and its application

**DOI:** 10.1007/s00410-024-02173-6

**Published:** 2024-09-30

**Authors:** Edgar Dachs, Artur Benisek

**Affiliations:** Department of Chemistry and Physics of Materials, Division Mineralogy, University of Salzburg, Jakob-Haringerstrasse 2a, 5020 Salzburg, Austria

**Keywords:** KFMASHTO biotite, Activity model, Thermodynamic mixing properties, Microscopic interaction parameters, Pseudosection, Metapelite

## Abstract

A new activity model for biotite is formulated in the system K_2_O-FeO-MgO-Al_2_O_3_-SiO_2_-H_2_O-TiO_2_-O_2_ (KFMASHTO), which extends that for the KFMASH system by introducing a titanium-biotite and a ferric-biotite end-member (*tbio*: K(TiMg_2_)[(O)_2_(AlSi_3_)O_10_] and *fbio*: K(Fe^3+^Mg_2_)[(OH)_2_(Al_2_Si_2_)O_10_]), as well as a pyrophyllite end-member (*pyp*: Al_2_[(OH)_2_Si_4_O_10_]) that accounts for the presence of octahedral excess-Al in natural biotites. Phonon calculations applying density functional theory (DFT) using the software *Castep* yielded the standard entropies of *tbio* and *fbio* as *S*^o^_tbio_ = 328.06 J/(mol·K) and *S*^o^_fbio_ = 301.69 J/(mol·K), and their heat capacity functions. From experimental phase-equilibrium data, the enthalpy of formation value of *tbio* was constrained as $\Delta {H}_{f,tbio}^{o}$ = −6124.68 ± 3.33 kJ/mol. Natural data were used to derive $\Delta {H}_{f,fbio}^{o}$= −5935.3 ± 6.6 kJ/mol. The single-defect DFT method was applied to parameterize important macroscopic mixing properties (macro-*W*’s) involving *tbio* and *pyp* end-members in the model (*fbio* was treated ideal). *Castep*-derived microscopic interaction energies (micro-*w*’s) are presented herein for KFMASH-biotite. The octahedral same-site (M1) Mg–Al mixing micro-*w* (*w*_MgAl(M1)_), the same-site tetrahedral Si-Al mixing parameter (*w*_SiAl(T1)_) and the related cross-site term are: *w*_MgAl(M1)_ = 82.5 kJ/mol, *w*_SiAl(T1)_ = 95.6 kJ/mol (two T1-sites) and ${w}_{MgAlAlSi(M1T1)}=$ 175.1 kJ/mol. The linear combination of these micro-*w*’s gives a macroscopic *W*_phleas_ = 18.8 kJ/mol, that is not transferable to other mineral groups. Micro *w*’s for Mg-Fe mixing in biotite (*w*_MgFe(M1)_, *w*_MgFe(M2),_ ${w}_{MgMgFeFe(M1M2)}$), are all close to ideality. The biotite activity model of this study is thus a first example of next-generation activity models that use DFT- and thus physically based micro-*w*’s and reassembled macro-*W*’s for petrological calculations. Test calculations on 5 samples from low- to high-grade metamorphic environments covering metapelite to greywacke bulk-compositions using *Perple_X* suite of programs illustrate the performance of the new biotite activity model. Computed mineral-chemistries are in all cases in better agreement with measured compositions than resulting from published activity models of biotite.

**Supplementary Information:**

The online version contains supplementary material available at 10.1007/s00410-024-02173-6.

## Introduction

For phase-equilibrium calculations involving biotite, an important phase especially in metamorphic (metapelite) assemblages, a thermodynamic model is a prerequisite in order to compute the activity-composition relationships for its constituent end-members. In petrological software, like *Perple_X* (Connolly 2005) and *Thermocalc* (Powell et al. 1998; Powell and Holland 2008) two activity models for biotite are currently in use, named Bi(W) and Bio(TCC) in *Perple_X*. The former, Bi(W), is a model for the K_2_O-FeO-MgO-Al_2_O_3_-SiO_2_-H_2_O-TiO_2_-O_2_ (KFMASHTO) system, as outlined in White et al. (2000, 2007, 2014), with KFMASH mixing parameters as originally calibrated by Powell and Holland (1999) and revised in Holland and Powell (2006). Tajčmanova et al. (2009 – T09) undertook a reformulation and reparameterization of this Bi(W)-model resulting in Bio(TCC). Both models are identical with regard to their KFMASH-parts, except that the enthalpy of the ordering reaction relating the biotite components annite (*ann*, KFe_3_[(OH)_2_AlSi_3_O_10_]), phlogopite (*phl,* KMg_3_[(OH)_2_AlSi_3_O_10_]) and ordered-Fe–Mg biotite (*obi,* KFeMg_2_[(OH)_2_AlSi_3_O_10_]), i.e., 2/3 *phl* + 1/3 *ann* = *obi*, was lowered by several kJ/mol in T09, affecting the octahedral Al-content predicted for KFMASH-biotite compared to Bi(W). Whereas in both models the ferric biotite end-member *fbio* is formulated identically, the recalibration of T09 is based on a differently defined Ti-biotite end-member *tbio*, with Ti replacing Mg on the M2 position and deprotonation of the hydroxyl site to achieve charge balance. Additionally, both models differ in their data extraction strategy and the underlying data set of ferric Fe- and Ti-contents of biotite from natural and experimental sources. Both models have been tested by Gervais et al. (2021), in their ability to reproduce the results of published partial melting experiments. According to their assessment, Bio(TCC) is the more favourable model and should be used in phase-equilibrium calculations.

Dachs and Benisek (2019, 2021a, b) recently presented a new biotite activity model for the KFMASH system. By applying an integrated approach combining experimental results from calorimetry, IR-spectroscopy and from phase-equilibrium studies with DFT calculations, they derived revised values for the thermodynamic standard state properties of the main biotite end-members *ann*, *phl* and eastonite (*eas,* KMg_2_Al[(OH)_2_Al_2_Si_2_O_10_]), as well as their mutual mixing behaviour. According to their analysis, near ideal activity-composition relationships are indicated for the *phl*-*ann* binary. Dachs et al. (2021b) constructed pseudosections for various KFMASH metapelites, where the performance of this model is compared to results obtained with Bi(W). They demonstrated that excess octahedral Al (Al^VI^_ex_) is present in non-negligible amounts in natural metapelite biotites (Dachs et al. 2021b, Fig. 12), and emphasized the necessity to account for this by incorporating an adequate component into existing biotite activity models. Bi(W) and Bio(TCC) are not able to model Al^VI^_ex_, because they are built of Tschermak substitution related biotite end-members only.

The aim of the present contribution is to

- provide a new biotite activity model for use in phase-equilibrium calculations by extending the KFMASH-model of Dachs and Benisek (2021a) to a more general Fe^3+^- and Ti-bearing metapelite system (KFMASHTO). This is achieved by a) introducing a new di-octahedral biotite end-member that allows modelling of Al^VI^_ex_ (*pyp*), b) incorporating a Fe^3+^- and a Ti-biotite end-member into this model (see Table 1 for definition of end-members and their site populations). This includes to determine the thermodynamic standard state properties and to parameterize relevant mixing properties for these new end-members, by performing DFT calculations combined with the evaluation of experimental data,

**Table 1:** Independent end-members and site distributions for KFMASHTO biotite. Note that the interlayer(A)-site in most endmembers is occupied by K (except *pyp* with a vacancy on A), and that the two T2-sites in all end-members are occupied by Si

	End-member	Formula	Site distribution				
			A	1 M1	2 M2	2 T1	2H
1	phl	KMg_3_[(OH)_2_(AlSi_3_)O_10_]	K	Mg	MgMg	AlSi	(OH)_2_
2	ann	KFe_3_[(OH)_2_(AlSi_3_)O_10_]	K	Fe	FeFe	AlSi	(OH)_2_
3	eas (ordered)	K(AlMg_2_)[(OH)_2_(Al_2_Si_2_)O_10_]	K	Al	MgMg	AlAl	(OH)_2_
4	obi (ordered)	K(FeMg_2_)[(OH)_2_(AlSi_3_)O_10_]	K	Fe	MgMg	AlSi	(OH)_2_
5	pyp	Al_2_[(OH)_2_Si_4_O_10_]	□^1)^	□^1)^	AlAl	SiSi	(OH)_2_
6	tbio	K(TiMg_2_)[(O)_2_(AlSi_3_)O_10_]	K	Mg	TiMg	AlSi	O_2_
7	fbio	K(Fe^3+^Mg_2_)[(OH)_2_(Al_2_Si_2_)O_10_]	K	Fe^3+^	MgMg	AlAl	(OH)_2_

^1)^ vacancy
- perform test calculations with *Perple_X* demonstrating the performance of the new model, Bio(D), compared to using the published models Bi(W) and Bio(TCC).

## Computational methods

### Density functional theory (DFT)

Quantum–mechanical calculations were based on the DFT plane-wave pseudopotential approach implemented in the *Castep* code (Clark et al. 2005) included in the Materials Studio software from Biovia®. The calculations used the local density approximation (LDA) for the exchange–correlation functional (Ceperley and Alder 1980). To describe the core-valence interactions, ultrasoft pseudopotentials were used with the 1s^1^, 2s^2^2p^4^, 2p^6^3s^2^, 3s^2^3p^1^, 3s^2^3p^2^, 3s^2^3p^6^4s^1^, 3s^2^3p^6^3d^2^4s^2^ and 3d^6^4s^2^ electrons explicitly treated as valence electrons for H, O, Mg, Al, Si, K, Ti and Fe, respectively. The calculations on Fe-containing minerals used the *LDA* + *U* approach (Zhou et al. 2004), with *U* = 2.0 – 4.0 eV applied to the d orbitals of Fe. The k-point sampling used a Monkhorst–Pack grid (Monkhorst and Pack 1976) with a spacing of 0.02 Å^−1^ for the energy calculations. Convergence was tested by performing calculations using a denser k-point grid. The structural relaxation was calculated by applying the BFGS algorithm (Pfrommer et al. 1997), where the maximum force on the atom was within 0.01 eV/Å.

The enthalpy of mixing was simulated by the single defect method (Sluiter and Kawazoe 2002), which investigates supercells with almost end-member composition having only a single substitutional defect. The energy calculations of the end-members and such supercells provide the interaction parameters, because the results can easily be transformed into the slopes of the heat of mixing function (Li et al. 2014). The transformation of *Castep* energies (in eV) into enthalpies (in kJ/mol) was done as outlined in Benisek and Dachs (2018, 2020).

Phonon calculations with *Castep*, with methods exemplified in Benisek and Dachs (2020), were used to compute the heat-capacity functions and standard entropies *S*^o^ for *tbio* and *fbio* end-members.

### Phase-equilibrium and pseudosection calculations

In order to extract Δ*H*^o^_f_ of *tbio* from experimental and in the case of *fbio* from natural data, self-written Mathematica programs were used to compute all required thermodynamic functions, based on biotite solution- and end-member properties given in Table 2 and Table 3. The standard state properties of all other phases, as well as their heat capacity, volume, thermal expansion and bulk modulus parameters were taken from Holland and Powell (2011) (*Thermocalc* file ‘tc-ds62.txt’). Solid solution properties, except that for biotite, were taken from White et al. (2014).

**Table 2:** Macroscopic mixing parameters *W* for KFMASHTO biotite (values in kJ/mol). Except for *ann*-*phl*, *W*’s are for symmetrical mixing. Mixing involving *fbio* is treated ideal (except mixing towards *pyp* with *W*_fbiopyp_ = 120 kJ/mol)

*W*	ann	eas	obi	tbio	pyp
phl	DB21: *W*_annphl_ = -8.8^1)^ *W*_phlann_ = 14.3^1)^	BD24: 19^2)^ DB19:	DFT-mw: -0.1	DFT-sd: 0	DFT-sd: 116.8
ann		DB21: -5	DFT-mw: -0.4	DFT-sd: -30	DFT-sd: 108.2
eas			DB21: -5	0^3)^	120^3)^
obi				0^3)^	120^3)^
tbio					120^3)^

^1)^ DB21: Dachs and Benisek (2021a)

^2)^ BD24: Benisek and Dachs (2024) derived from density functional calculations using *Castep* applying the single-defect method (DFT-sd) and DFT-calculations on micro-*w*’s (DFT-mw, see Tab. 5); this value is in accordance with the Δcorr-derived *W*_phleas_ in Dachs and Benisek (2019 – DB19).

^3)^ assumed value

**Table 3:** Standard state (1 bar, 298.15 K) thermodynamic properties of biotite end-members. Values for *ann*, *phl* and *eas* are from Dachs and Benisek (2021a), those for *tbio* and *fbio* are from this study (*pyp* data are not given but are thermodynamic dataset values from *Perple_X*-file hp62ver.dat).* C*_p_ = k_0_ + k_1_∙*T* ^−0.5^ + k_2_∙*T* ^−2^ + k_3_∙*T* ^−3^ in J/(mol·K)

	Δ*H*^o^_f_	*S*^o^	*V*^o^	k_0_	k_1_	k_2_·10^–7^	k_3_·10^–9^
	kJ/mol	J/(mol·K)	J/(mol·bar)				
ann	−5131.55 ± 2.34	422.9 ± 2.9	15.48	728.6	−5581	−0.2896	0.2957
phl	−6209.87 ± 0.50	330.9 ± 2.2	14.96	667.4	−3914	−1.5240	2.1727
eas	−6352.00 ± 3.72	294.5 ± 3.0	14.65	656.9	−3622	−1.7098	2.3180
tbio	−6131.11 ± 3.33^1)^	328.06^2)^	14.81	594.1	−2690	−1.8837	2.7692
fbio	−5933.63 ± 6.55^3)^	301.69^2)^	15.41	642.0	−3034	−1.5387	1.8390

^1)^ extracted from the experimental data of PD93.

^2)^ derived from DFT calculations using *Castep*. *S*^o^ already includes configurational entropy contributions. Note: molar volume and *C*_p_ coefficients for *tbio* and *fbio* are also from DFT calculations, as well as bulk modulus *bm* for *tbio*: *bm* = 63.3 + 9.08*P(kbar). Thermal expansion coefficients for *tbio* and *fbio* are those of *ann* from the thermodynamic database.

^3)^ extracted from natural data of Williams and Grambling (1990).

The standard state data of biotite end-members were then implemented into the *Perple_X* data file ‘hp62ver.dat’, the solution properties as biotite activity model ‘Bio(D)’ into the *Perple_X* file ‘solution_model.dat’ (see supplementary Tables 1a and 1b). The other *Perple_X* solution models used were: Chl(W), Opx(W), Gt(W) Mica(W), St(W), Crd(W), Ilm(WPH), Mt(W), Sp(WPC), ‘feldspar’, melt(W), for chlorite, orthopyroxene, garnet, mica, staurolite, cordierite, ilmenite, magnetite, spinel, feldspar solid-solutions and melt, respectively.

Compositional data of biotite in various assemblages (like Al^IV^-, Al^VI^-, Al^VI^_ex_-, Ti- contents and X_Fe_), were computed with the *Perple_X* program *Werami* combined with self-written Mathematica^®^ functions to extract mineral-chemical parameters of biotite for predefined values of P and T from data files generated with *Werami.* For comparison with results obtained by using Bio(D), calculations were repeated with the same set of solution models but with Bi(W) or Bio(TCC) as activity model for biotite. In order to simulate logf_O2_-conditions defined by oxygen-buffers like NNO (nickel—nickel oxide), QFM (quartz-fayalite-magnetite), CCO (cobalt-cobalt oxide) or CCH4 (graphite-methane), the file ‘NNO_PerpleX_fO2.xls’ was used (provided by St. Roozen and V. van Hinsberg, Montreal). By adding Ni and O_2_ to the list of components in a *Perple_X* calculation and changing the entropy of Ni to an appropriate value, as computed with this Excel sheet, logf_O2_-conditions, prevailing either during experimentation or during natural metamorphic events, could be simulated. 

Pseudosections for the test-samples were also computed with *Perple_X* and the above-mentioned data files and solution models, raw versions are given in Online Resource 7. Isopleths of mineral-chemical parameters were generated with the program *PyWerami* (written by O. Lexa, Czech Geological Survey, see https://pypi.org/project/pywerami/).

## Results

### A new activity model for KFMASHTO biotite

The activity model of biotite presented in this study is an extension of the KFMASH model of Dachs and Benisek (2021a – DB21). The same logic as described there is applied here to extend it for the new components *tbio*, *fbio* and *pyp* (Table 1). As a simplification, disordered eastonite is no longer considered, as it was shown in DB21 that Mg–Al mixing on M2 is negligible at metamorphic P–T conditions.

#### Titanium-biotite end-member (tbio)

To calibrate the *tbio* end-member, we followed the strategy to first compute *C*_p_ and the standard-entropy, *S*^o^_tbio_, from *Castep*-calculations with methods exemplified in Benisek and Dachs (2020). Fitting the so obtained super-ambient *C*_p_ to a Berman-Brown-type polynomial (Berman and Brown 1985) gave:

1 $$C_{{\text{p}}}^{{{\text{tbio}}}} ({\text{J}}/\left( {{\text{mol}}\cdot{\text{K}}} \right))\, = \,{594}.{1} - {269}0 \cdot T^{{ - 0.{5}}} - {1}.{8837} \cdot {1}0^{{7}} \cdot T^{{ - {2}}} \, + \,{2}.{7692} \cdot {1}0^{{9}} \cdot T^{{ - {3}}}$$

By numerically integrating the *Castep*-computed *C*_p_/T of *tbio* over the range 0 to 298.15 K, its *S*^o^ was constrained as *S*^o^_tbio_ = 328.06 J/(mol·K) (Table 3).

Following Tajčmanova et al. (2009), we then extracted Δ*H*^o^_f_ values of the *tbio* end-member based on the equilibrium (mineral abbreviations not yet defined are: *ilm* = ilmenite, *qtz* = quartz, *py* = pyrope, *alm* = almandine):

2 $${\text{phl}}\, + \,{\text{3 eas}}\, + \,{\text{6 ilm}}\, + \,{\text{12 qtz}}\, = \,{\text{1 py}}\, + \,{\text{2 alm}}\, + \,{\text{6 tbio}}\, + \,{\text{6 H}}_{{2}} {\text{O}}$$

from experimental data of Patino-Douce et al. (1993 – PD93, see compilation in supplementary Table 2). The experimental data of Vielzeuf and Montel (1994 – VM94), and of Patino-Douce and Beard (1995 – PDB95), as well as the natural data of Williams and Grambing (1990 – WG90) and Holdaway et al. (1997 – H97) were also considered, but not used for extraction purposes for reasons discussed below.

In terms of chemical potentials μ_i_, which are given by ${\mu }_{i}={\mu }_{i}^{o}+RTln{a}_{i}$, where μ_i_^o^ is the chemical standard potential (i.e., the Gibbs free energy of pure i at *P* and *T*), *R* the gas constant and *a*_i_ the activity of component i in a solid solution, it follows for equilibrium (2) that:

3 $${\mu }_{tbio}=\left(\frac{1}{6}\right)\left(3{\mu }_{phl}+3{\mu }_{eas}+6{\mu }_{ilm}+12{\mu }_{qtz}-1{\mu }_{py}-2{\mu }_{alm}-6{\mu }_{H2O}\right)$$

Writing ${\mu }_{tbio}^{o}={\mu }_{tbio}-RTln{a}_{tbio}$ at *P* and *T* in terms of standard thermodynamic properties, i.e. enthalpy of formation, $\Delta {H}_{f,tbio}^{o}$, entropy, ${S}_{tbio}^{o}$, volume, *V*_tbio_ and heat capacity, *C*_p_:

4 $${\mu }_{tbio}^{o}={G}_{tbio}^{P,T}={\Delta {H}_{f,tbio}^{o}+\underset{298.15}{\overset{T}{\int }}{C}_{P,tbio}dT}-T\left({S}_{tbio}^{o}+\underset{298.15}{\overset{T}{\int }}\frac{{C}_{P,tbio}}{T}dT\right)+\underset{1}{\overset{P}{\int }}{V}_{tbio}dP$$

and rearranging gives:

5 $${\mu }_{tbio}^{o}-\underset{298.15}{\overset{T}{\int }}{C}_{P,tbio}dT+T\left({S}_{tbio}^{o}+\underset{298.15}{\overset{T}{\int }}\frac{{C}_{P,tbio}}{T}dT\right)-\underset{1}{\overset{P}{\int }}{V}_{tbio}dP= \Delta {H}_{f,tbio}^{o}+{T(-S}_{tbio}^{o})$$

**Fig. 1:**
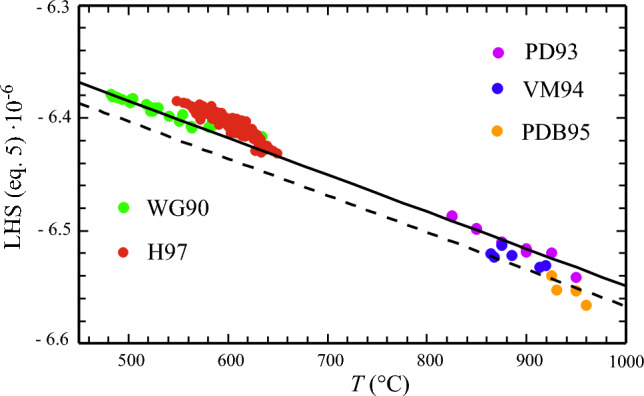
Left hand side (LHS)*10^–6^ of Eq. (5) plotted against temperature in (°C) showing experimental (T > 800 °C) and natural data (T < 650 °C) relevant for the *tbio* end-member. Magenta: Experimental data (with ilmenite as Fe-Ti oxide phase) of Patino Douce et al. (1993 – PD93) using a natural peraluminious metapelite bulk composition, blue: Vielzeuf and Montel (1994 – VM94) using a quartz-rich meta-greywacke bulk and brown: Patino Douce and Beard (1995 – PDB95) using a synthetic biotite gneiss bulk composition. Green: Natural metapelite data of Williams and Grambling (1990 – WG90) and red: Holdaway et al. (1997 – H97). The solid line was computed with the *Castep*-derived ${S}_{tbio}^{o}$ = 328.06 J/(mol·K) and $\Delta {H}_{f,tbio}^{o}$ = −6131.11 kJ/mol, as extracted from PD93-experiments, broken line was computed using the same ${S}_{tbio}^{o}$ and $\Delta {H}_{f,tbio}^{o}$ = −6149.44 kJ/mol, as would result from PDB95-experiments

Equation (5) yields a straight-line relationship (Fig. 1), if its left-hand-side (LHS) is plotted against temperature with a slope of ${-S}_{tbio}^{o}$. By using the *Castep*-derived ${S}_{tbio}^{o}$, $\Delta {H}_{f,tbio}^{o}$ can be directly extracted from available data using Eq. (5). Quartz and H_2_O were treated as pure phases and the experimentally measured compositions of garnet, ilmenite and biotite were used to compute the required activities of the garnet, ilmenite and biotite end-members (activity models used in *Perple_X* and *Mathematica* calculations were Grt(W) for garnet, Ilm(WPH) for ilmenite and Bio(D), the new biotite mixing model from this study with mixing parameters given in Table 2). The macroscopic *W*’s describing mixing between Ti and either Mg or Fe were constrained from *Castep*-calculations using the single-defect method and are *W*_phltbio_ =  ~ 0 kJ/mol, i.e., close to ideality, and *W*_anntbio_ = -30 kJ/mol. The less important *W*’s, namely *W*_eastbio_ and *W*_obitbio_ were set to zero.

**Fig. 2:**
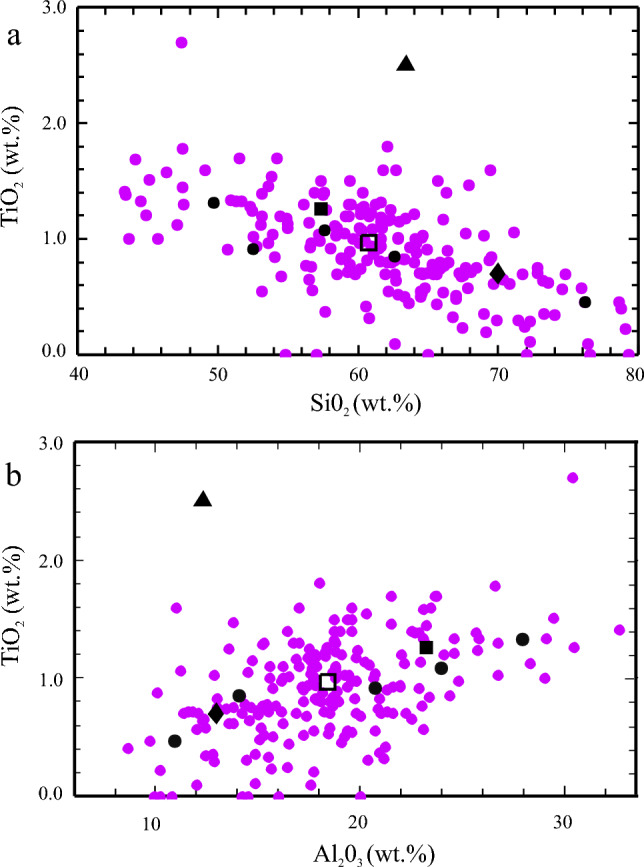
**a)** Bulk-SiO_2_ vs. bulk-TiO_2_ **b)** bulk-Al_2_O_3_ vs. bulk-TiO_2_ (in wt.%) from the metapelite database of Forshaw and Pattison (2021 – magenta dots, n = 217, mean plotted as open square), compared to the bulks used in the experiments of Patino Douce et al. (1993, filled square), Vielzeuf and Montel (1994, filled diamond) and of Patino-Douce and Bird (1995, filled triangle). Black dots represent compositions of the five test samples (supplementary Table 5)

Using the experimental PD93 data, performed on a peraluminious metapelite bulk-composition, the derived Δ*H*^o^_f_ of *tbio* from Eq. (5) is -6131.11 ± 3.33 kJ/mol (Table 3). The PD93 data have been preferred over that from VM94 and PDB95, because a) they comprise the most comprehensive experimental data set, not only providing mineral-chemical analyses of biotite and garnet from the experimental charges, but also of ilmenite, b) least-square calculations showed that with the biotite end-members chosen herein (Table 1), the experimental biotite compositions reported in PD93 could be reproduced with much less residuals than was the case for VM94 or PDB95 biotite data, and c) the bulk composition used in PD93 experiments (HQ-36, supplementary Table 5) best matches that of natural metapelites, as it plots relatively close to the metapelite-average of Forshaw and Pattison (2021 – FP21). On the other hand and shown in Fig. 2a and b, the metagreywacke composition in VM94 experiments (sample CEVP) is much more Si-rich and the synthetic biotite gneiss in PDB95 experiments (sample SBG, crushed from a quartz-diorite, supplementary Table 5) lies off all bulk compositions reported for natural metapelites and greywackes with a markedly larger bulk-TiO_2_ content (Fig. 2).

#### Ferric-biotite end-member (fbio)

Phonon-calculations with *Castep* on the ferric-biotite end-member and the processing of these data following Benisek and Dachs (2020) and fitting to a Berman/Brown-type *C*_p_-polynomial yielded:

6 $$C_{{\text{p}}}^{{{\text{fbio}}}} ({\text{J}}/\left( {{\text{mol}}\cdot{\text{K}}} \right))\, = \,{642}.0 - {3}0{34} \cdot T^{{ - 0.{5}}} - {1}.{5387} \cdot {1}0^{{7}} \cdot T^{{ - {2}}} \, + \,{1}.{839}0 \cdot {1}0^{{9}} \cdot T^{{ - {3}}}$$

The standard entropy of *fbio* was determined as *S*^o^_fbio_ = 301.69 J/(mol·K) from the *Castep* phonon-calculations (Table 3) and its $\Delta {H}_{f}^{o}$ ranging between -5888.6.0 kJ/mol and -5897.3 kJ/mol, for U = 2.5 (default value in *Castep*) and U = 4.0 eV, respectively. For comparison, the natural mineral-chemical data of biotite and garnet provided by Williams and Grambling (1990), who studied metapelitic rocks from Proterozoic rocks of northern New Mexico, were also used to extract $\Delta {H}_{f,fbio}^{o}$. These authors also determined the ferric iron content in these minerals by Mössbauer spectroscopy, wet-chemical analysis or by the composition of coexisting oxides in their samples.

From the reaction (abbreviations not yet defined: *sil* = sillimanite, *ky* = kyanite, *mt* = magnetite):

7 $${\text{fbio}}\, + \,{\text{1 alm}}\, + \,{\text{2 sil}}/{\text{ky}}\, = \,{\text{6 eas}}\, + \,{\text{5 qtz}}\, + \,{\text{3 mt}}$$

$\Delta {H}_{f,fbio}^{o}$ was then computed in a similar manner as described above for *tbio* (*mt*, *qtz*, *sil* and *ky* were treated as pure phases, and *fbio* interactions with the other biotite end-members assumed to be ideal). This yielded $\Delta {H}_{f,fbio}^{o}$= -5933.6 ± 6.6 kJ/mol (Table 3), around 40 kJ/mol different from the *Castep*-derived values, which is a deviation of ~ 0.7%. We decided to adopt the more negative $\Delta {H}_{f}^{o}$ value in concurrence with ideal *fbio* interactions.

#### Pyrophyllite end-member (pyp)

**Fig. 3:**
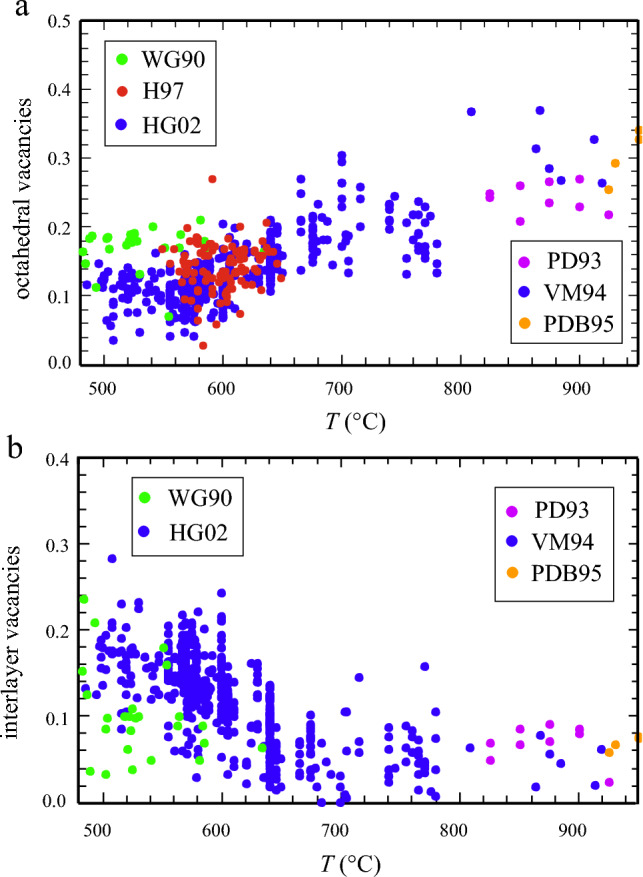
**a)** Octahedral vacancies, ^VI^□, in metapelite biotites from natural sources and from experimental studies on Ti-bearing natural biotite (points > 800 °C). Following Labotka (1983) ^VI^□ was computed as: ^VI^□ = 3 – (Mg + Fe + Fe^3+^  + ^VI^Al + Ti). Natural sources: Green: Williams and Grambling (1990 – WG90), n = 25; red: Holdaway et al. (1997 – H97), n = 98; dark-blue: Henry and Guidotti (2002 – HG02), Main biotite data set (n = 562). Experimental sources: Magenta: Patino Douce et al. (1993 – PD93); blue: Vielzeuf and Montel (1994 – VM94); brown: Patino Douce and Beard (1995 – PDB95). **b)** Interlayer-vacancies, ^A^□, in metapelitic biotites from natural sources and from experimental studies using Ti-bearing natural biotite (points > 800 °C). Following Labotka (1983) ^A^□ was computed as: ^A^□ = 1 + ^VI^Al + Fe^3+^  + 2Ti – ^IV^Al – 2^VI^□). Natural and experimental sources as in a)

It is well known that natural biotites from metapelitic rocks have octahedral Al-contents in excess of that balanced by the Tschmak substitution, i.e. by tetrahedral-Al minus 1 (see e.g. literature review in Guidotti 1984, or Fig. 12 in Dachs and Benisek 2021b). As noted by Guidotti (1984) and displayed in Fig. 3a, octahedral cations in biotite show an universal deficiency, in the order of 0.05 to 0.2 apfu (atoms per formula unit), to the ideal value of 3.0 apfu (for a formula based on 11 oxygens). The interlayer site also typically contains less cations than expected (1.0), with values around 0.9 apfu being most common (Fig. 3b).

We thus decided to introduce a di-octahedral pyrophyllite component (*pyp*), ^[A]^□^[M1]^□^[M2]^Al_2_[(OH)_2_^[T1]^Si_2_^[T2]^Si_2_] to account for this. The *pyp* component generates vacancies in the interlayer position, as well as vacancies on M1 and excess-Al on M2 based on the exchange:

8 $$^{{\left[ {\text{A}} \right]}} {\text{K}}^{ + } \, + \,^{{[{\text{M1}}]}} {\text{Mg}}^{{{2} + }} \, + \,{2}^{{[{\text{M2}}]}} {\text{Mg}}^{{{2} + }} \, + \,^{{[{\text{T1}}]}} {\text{Al}}^{{{3} + }} \, = \,^{{[{\text{A}}]}} \square \, + \,^{{[{\text{M1}}]}} \square \, + \,{2}^{{[{\text{M2}}]}} {\text{Al}}^{{{3} + }} \, + \,^{{[{\text{T1}}]}} {\text{Si}}^{{{4} + }}$$

which is obtained by subtracting the *phl*- from the *pyp*-composition. This exchange represents a solution of tri-octahedral biotite towards the di-octahedral sheet silicate *pyp* and is preferred over e.g. using a muscovite-component to model excess octahedral-Al in biotite.

Our *Castep* calculations using the single-point defect method indicate strongly positive interaction parameters over 100 kJ/mol for mixing of the main biotite components *phl* and *ann* towards *pyp* (*W*_phlpyp_ = 116.8 kJ/mol, *W*_annpyp_ = 108.2 kJ/mol). Based on these results all remaining *W*’s involving *pyp* were set to 120 kJ/mol (Table 2).

### Microscopic biotite interaction parameters from Castep calculations

In the context of activity coefficient expressions for multi-site solid solution, it was shown by Powell and Holland (1993 – PH93) that so-called ‘*macroscopic*’ interaction parameters *Wij*, appearing in these expressions (Eq. (20) in HP93), describing symmetric mixing between two end-members *ij *in a solid solution (e.g., *W*_phlann_), can be written as specific linear combinations of ‘*microscopic*’ same-site and cross-site interaction parameters *w* (eqs. (7) and (16) in PH93) that relate to mixing of elements on and between sites. This is the basis of the ‘*micro-*Φ’-approach of Powell et al. (2014), where micro-*w*’s are approximated from systems where good data exist. Using heuristic arguments, these are then transferred to mineral groups with little or no data and reassembled in order to allow parameterization of macroscopic *W*’s for these systems. As an example, *w*_FeMg,oct_, i.e., same-site mixing of Fe and Mg on an octahedral site, is taken to be 4 kJ per exchange based on experimental data for Fe–Mg olivine (Hackler and Wood 1989; Wiser and Wood 1991). Following this procedure and assuming cross-site terms to be negligible, macroscopic *W*_annphl_ would then be given by *W*_annphl_ = 3*w*_FeMg(oct)_ = 12 kJ/mol in the case of biotite (e.g. Holland and Powell 2006), a value used in the biotite activity models Bi(W) and Bio(TCC).

Recently, Benisek and Dachs (2024 – BD24) applied DFT methods to compute enthalpic microscopic interaction parameters for petrologically important substitutions (Mg–Al, Si-Al, Mg-Ti, Mg-Ca and Mg-Fe) for a variety of mineral groups. As shown in this work, micro-*w*’s correlate with the ‘oxygen-packing fraction’ (OPF) of solid-solutions, whereby this parameter may be envisaged as a kind of density of oxygen in the unit-cell. OPF in turn correlates with the bulk-modulus (stiffness) of minerals. Micro-*w*’s are thus in principle not interchangeable between mineral groups and BD24 demonstrated that e.g. the micro-*w* for Mg–Al mixing in biotite is less than half that in diopside-cats pyroxene.

In the following, we present *Castep*-derived micro-*w*’s for KFMASH-biotite in more detail.

#### Phlogopite-annite join

*Castep* computations in this join, due to the presence of iron, were done in spin-polarized mode for Fe and are complicated by the fact that the so-calculated energies are dependent on the choice of the parameter U (2.5 eV by default). We thus performed several calculations for each required biotite composition using U values of 1.0, 2.0, 2.5 and 3.0 and the energies given in Table 4 are means of these values. The decomposition of macroscopic *W*_phlann_ into microscopic *w*’s is (supplementary Table 4):

9 $$W_{phlann} = w_{{MgFe\left( {M1} \right)}} + w_{{MgFe\left( {M2} \right)}} + w_{{MgMgFeFe\left( {M1M2} \right)}}$$

**Table 4:** *Castep* energies for various mineral phases of the *ann-phl* and the *phl-eas* joins, used to compute microscopic interaction parameters *w*’s for these binaries, as given in Tab. 5. *Castep* was run in the LDA non-conserving mode for the *phl*–*eas* join and in spin-polarized mode for the *ann-phl* binary using ultrasoft pseudopotentials. The values given for each composition are means of 4 calculations with U = 1, 2, 2.5 and 3

Join i-j	Mineral phase	Abbreviation	Site population			Charge	Castep energy ε kJ/mol
			1 M1	2 M2	2 T1		
ann-phl	annite	ann	Fe	FeFe	AlSi	0	−871,236.96
	phlogopite	phl	Mg	MgMg	AlSi	0	−905,636.00
	ordered Fe–Mg biotite	obi	Fe	MgMg	AlSi	0	−894,172.07
	Fe^M2^(ordered biotite)	Fe^M2^(obi)	Fe	MgFe	AlSi	0	−882,704.71
	Mg_0.5_^M1^(ordered biotite)	Mg_0.5_^M1^(obi)	Mg_0.5_Fe_0.5_	MgMg	AlSi	0	−899,904.09
phl-eas	phlogopite	phl	Mg	MgMg	AlSi	0	−1,078,178.27
	eastonite (ordered)	eas	Al	MgMg	AlAl	0	−925,453.00
	Al^M1^-phlogopite	Al^M1^(phl)	Al	MgMg	AlSi	+ 1	−930,990.39
	Al^T1^-phlogopite	Al^T1^(phl)	Mg	MgMg	AlAl	−1	−1,072,816.00
	Al_0.5_^M1^-phlogopite	Al_0.5_^M1^(phl)	Mg_0.5_Al_0.5_	MgMg	AlSi	+ 1/2	−1,004,563.71
	Al_1.5_^T1^-phlogopite	Al_1.5_^T1^(phl)	Mg	MgMg	Al_1.5_Si_0.5_	−1/2	−1,075,473.24

In summary, the micro *w*’s relevant for Mg-Fe mixing in biotite are all close to ideality, i.e., they have only small negative values that are zero within error. The least dependence on U (and thus smallest uncertainty) shows *w*_MgFe(M1)_ with a value of -0.1 ± 0.8 kJ/mol. Larger uncertainties are indicated for *w*_MgFe(M2)_ = -0.4 ± 3.6 kJ/mol and *w*_MgMgFeFe(M1M2)_ = -1.9 ± 2.7 kJ/mol. The reassembled *W*_phlann_ is -2.4 ± 5.0 kJ/mol, in reasonable agreement with the Fe–Mg mixing behaviour derived by Dachs and Benisek (2021a) from experimental data, i.e. weak negative deviation from ideality for Fe-rich and weak positive deviation for Mg-rich biotites, as modelled by the two Margules parameters *W*_phlann_ = 14.3 kJ/mol and *W*_annphl_ = -8.8 kJ/mol (Table 2). From the decomposition of macro-*W*’s, it then follows that *W*_phlobi_ = *w*_MgFe(M1)_ and *W*_annobi_ = *w*_MgFe(M2)._

#### Phlogopite-eastonite join

For macroscopic *W*_phleas_, the decomposition into corresponding micro-*w*’s is (e.g., Powell et al. 2014; Benisek and Dachs 2024; see supplementary Table 4):

10 $$W_{phleas} = \left( \frac{1}{4} \right)\left( {4w_{{MgAl\left( {M1} \right)}} + w_{{SiAl\left( {T1} \right)}} - 2w_{{MgAlAlSi\left( {M1T1} \right)}} } \right)$$

The cross-site term ${w}_{MgAlAlSi(M1T1)}$, describing interactions of Mg, Al and Si between M1 and T1 sites in biotite represents the energy of the reciprocal reaction (e.g., Powell 1977, 1983; Powell et al. 2014; Wood and Nichols 1978; Chatterjee 1991):

11 $${\text{Mg}}^{{{\text{M1}}}} {\text{Al}}^{{{\text{T1}}}} \, + \,{\text{Al}}^{{{\text{M1}}}} {\text{Si}}^{{{\text{T1}}}} \, = \,{\text{Mg}}^{{{\text{M1}}}} {\text{Si}}^{{{\text{T1}}}} \, + \,{\text{Al}}^{{{\text{M1}}}} {\text{Al}}^{{{\text{T1}}}}$$

or written in terms of end-member energies:

12 $$w_{{MgAlAlSi\left( {M1T1} \right)}} = \varepsilon_{{\left( {phl} \right)}} + \varepsilon_{{\left( {east} \right)}} - \varepsilon_{{Al^{T1} \left( {phl} \right)}} - \varepsilon_{{Al^{M1} \left( {phl} \right)}}$$

Based on the *Castep*-computed energies (Table 4), cross-site ${w}_{MgAlAlSi(M1T1)}=$ 175.1 kJ/mol. With same-site *w*_MgAl(M1)_, = 82.5 kJ/mol and *w*_SiAl(T1)_ = 95.6 kJ/mol from Benisek and Dachs (2024), the recombination of same-site and cross-site micro-*w*’s according to Eq. (10) then gives a macroscopic *W*_phleas_ = 18.8 kJ/mol (Table 5), in good agreement with that resulting from calorimetry (Circone and Navrotsky 1992) or line-broadening in IR spectra (Dachs and Benisek 2019).

**Table 5:** Microscopic interaction parameters *w* of the biotite binaries *ann*-*phl* and *phl*-*eas*, calculated from *Castep* energies listed in Table 4 (the values given apply for 1 M1 site, 2 M2 and 2 T1 sites). A much more comprehensive set of micro-*w*’s for various substitutions in petrologically relevant mineral groups is given in Benisek and Dachs (2024). The linear combinations of micro-*w*’s to obtain macro-*W*’s (given in **bold face**), i.e., *W*_phleas_ and *W*_annphl_, are also listed (the full set of micro-*w*’s for the biotite activity model of this study can be found in supplementary Table 4)

Binary	Mixing type	Symbol	Mode of calculation (from *Castep* energies in Tab. 4)	Micro-*w* / macro-*W* (kJ/mol)
ann-phl	same-site	*w*_MgFe(M1)_	2Mg_0.5_^M1^(obi) ^1)^ – phl—obi	−0.1 ± 0.8
	same-site	*w*_MgFe(M2)_	2 Fe^M2^(obi) – obi – ann	−0.4 ± 3.6
	cross-site	*w*_MgMgFeFe(M1M2)_	(Fe^M2^(obi) + obi – phl – ann)	−1.9 ± 2.7
	macroscopic	***W***_**phlann**_	*w*_MgFe(M1)_ + *w*_MgFe(M2)_ + *w*_MgMgFeFe(M1M2)_	−**2.4**^2)^ ± 5.0
phl-eas	same-site	*w*_MgAl(M1)_	2Al_0.5_^M1^(phl) – phl – Al^M1^(phl)	82.5
	same-site	*w*_SiAl(T1)_	2Al_1.5_^T1^(phl) – phl – Al^T1^(phl)	95.6
	cross-site	*w*_MgAlAlSi(M1T1)_	(phl + eas – Al^T1^(phl) – Al^M1^(phl))	175.1
	macroscopic	***W***_**phleas**_	(1/4)(4*w*_MgAl(M1)_ + *w*_SiAl(T1)_ – 2*w*_MgAlAlSi(M1T1)_)	**18.8**^2)^, **19.0** ± 3.0^3)^, **25.4**^4)^

^1)^ see Tab. 4 for meaning of acronyms

^2)^ macroscopic *W*, calculated from *Castep*-derived micro-*w*’s listed above in the same column

^3)^ macroscopic *W*, calculated directly using *Castep* by applying the single-defect method (Benisek and Dachs 2020, 2024)

^4)^ experimental value of *W*, compatible with calorimetry (Circone and Navrotsky 1992; see also Dachs and Benisek 2019, 2021a, b)

## Discussion

### Titanium-biotite and ferric-iron biotite end-members (*tbio* and *fbio*)

The *Castep*-derived *S*^o^_tbio_ = 328.06 J/(mol·K) yields a slope of reaction (2) that is consistent with the experimental, as well as natural data, i.e., the experimental data of PD93 measured at high temperatures on a peraluminous pelite bulk composition (Fig. 1, dots in magenta) line up with the natural metapelite data of WG90 and H97 that represent metamorphic temperatures between ~ 500 and ~ 650 °C (Fig. 1, dots in green and red). The reasons for preferring the PD93 over the VM94 and PDB95 experimental data to extract $\Delta {H}_{f,tbio}^{o}$ have been discussed before. If the latter data were used, $\Delta {H}_{f,tbio}^{o}$ would amount to -6149.44 ± 5.95 kJ/mol, compared to $\Delta {H}_{f,tbio}^{o}$= -6131.11 ± 3.33 kJ/mol kJ/mol, as resulting from the preferred PD93 experiments. Caution is nevertheless indicated when applying Bio(D) to high-Ti systems, where the use of the former value may be more appropriate.

To compute $\Delta {H}_{f,tbio}^{o}$ with *Castep*, we used four different structural configurations, two representing local-charge balance (LCB) in the *tbio* end-member and two where the LCB criterion was released, i.e. allowing Ti also to reside on sites more distant to Mg than in the strict LCB situation – probably a more realistic scenario. The resulting $\Delta {H}_{f,tbio}^{o}$ = −6129 kJ/mol is in good agreement with the value extracted from PD93 experiments.

**Fig. 4:**
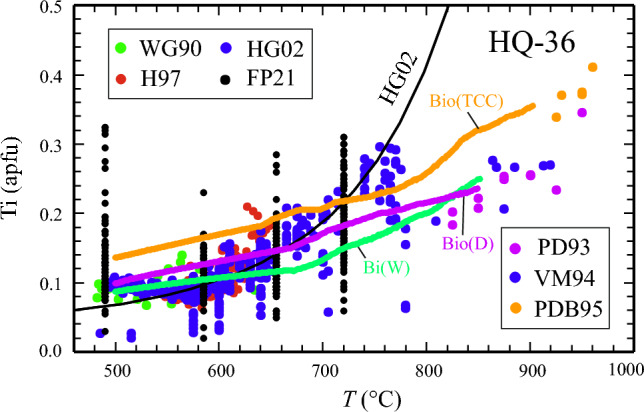
Ti-contents of biotite in metapelites (atoms per formula unit – apfu based on 11 oxygens) as function of temperature from experimental studies (points > 800 °C) and natural sources, compared to *Perple_X*-computed values. Experimental sources: Magenta: Patino Douce et al. (1993 – PD93); blue: Vielzeuf and Montel (1994 – VM94); brown: Patino Douce and Beard (1995 – PDB95). Natural sources: Green: Williams and Grambling (1990 – WG90), n = 25; red: Holdaway et al. (1997 – H97), n = 98; dark-blue: Henry and Guidotti (2002 – HG02), Main biotite data set (n = 562), black: Forshaw and Pattison (2021 – FP21), n = 452. The following temperatures were arbitrarily assigned to their metamorphic zone classification: grt: 490 °C, crd/st/and/ky: 585 °C, sil: 655 °C, kfs: 720 °C. Therefore their data appear vertically aligned. *Perple_X*-computed Ti-contents of biotite using the activity model Bio(D) and thermodynamic properties of biotite end-members from this study for peraluminous metapelite bulk-composition HQ-36, as used in the experiments of PD93, is shown as thick line in magenta (P = 7 kbar). For comparison, similar calculations with Bi(W) and Bio(TCC) and thermodynamic dataset values for biotite are also shown (green and brown thick lines). Black convex line is the biotite Ti-saturation surface according to Henry et al. (2005)

Figure 4 shows the above discussed experimental data and a bunch of natural biotite data in a plot of Ti apfu vs. temperature compared to calculated Ti contents for bulk HQ-36 at P = 7kbar (PD93 experiments) using *Perple_X*. With the biotite activity model Bio(D) and thermodynamic properties of biotite end-members from this study (Tables 2 and 3), biotite is stable in various assemblages between ca. 500 °C and 850 °C. At the low-temperature end Ti-contents in biotite are around 0.1 apfu in accordance with the gros of natural data, increasing steadily to values of ~ 0.2 apfu as present in PD93 experiments performed in the range 800 to 850 °C (Fig. 4, line in magenta). A similar behaviour is obtained using Bi(W) (Fig. 4, green line), whereas Bio(TCC) predicts systematically by 0.05–0.08 apfu larger Ti-contents (Fig. 4, dark-yellow line).

In case of the *fbio* end-member, a direct test of calculated vs. naturally observed Fe^3+^ content in biotite is not possible, because no bulk-rock compositions were given by WG90 for their samples, but just mineral-chemical data. Instead, we computed the Fe^3+^ content in biotite for PD93 experiments with *Perple_X*, implying redox conditions of the QFM buffer, f_O2_ conditions that supposedly prevailed during their experiments. The values obtained indicate low Fe^3+^ contents around 0.02 apfu, compared to the estimated values of PD93 that range between 0 and 0.07 apfu Fe^3+^ (supplementary Table 2).

#### Pyrophyllite end-member (pyp): Predicted vs. observed Al^IV^-, Al^VI^- and excess-Al^VI^-contents in biotite

**Fig. 5:**
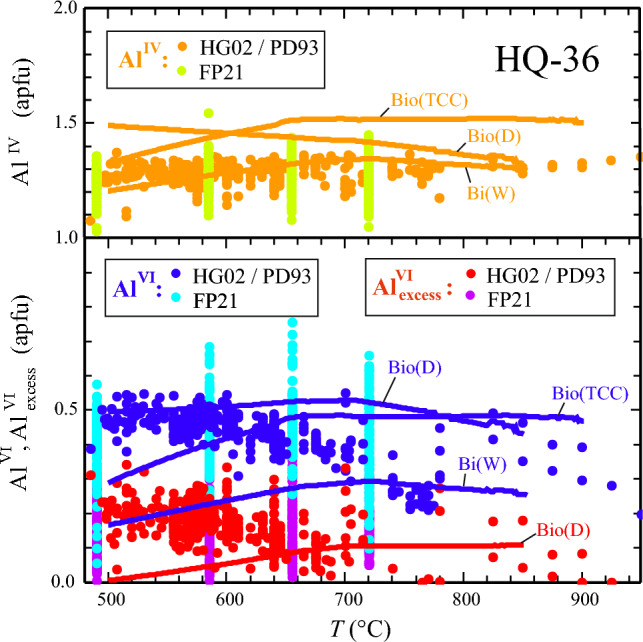
Al^IV^ -, Al^VI^- and Al^VI^_ex_-contents of biotite in metapelites (apfu based on 11 oxygens) as function of temperature from natural and experimental sources, compared to *Perple_X*-computed contents for the peraluminous metapelite bulk-composition HQ-36. Data < 800 °C are from natural sources: Al^IV^-, Al^VI^- and Al^VI^_ex_-contents from Main biotites (Henry and Guidotti 2002, – HG02, n = 562) are shown as brown, dark-blue and red points, corresponding values from the Forshaw and Pattison (2021 – FP21) biotite data set (n = 452) as light-green, light-blue and magenta points. The points > 800 °C are the experimental data of Patino Douce et al. (1993 – PD93). *Perple_X*-computed contents are drawn as thick lines, in brown for the Al^IV^-, in dark-blue for the Al^VI^- and in red for the Al^VI^_ex_-contents of biotite at P = 7 kbar. Biotite activity models used were Bio(D), in combination with thermodynamic properties of biotite end-members from this study, Bi(W) and Bio(TCC) with thermodynamic dataset values for biotite end-members. Note that for Al^VI^_ex_ only one curve, computed with Bio(D), exists (red)

In Fig. 5, the observed Al^IV^-, Al^VI^- and excess-Al^VI^-contents of over 1000 natural biotites are plotted as function of temperature using the Main biotite data set (Henry and Guidotti 2002), and the Forshaw and Pattison (2021) biotite data set. The former were collected from peraluminous metapelites that equilibrated between 4 and 6 kbar with a bulk composition probably similar or close to that as used by PD93 in their experiments (sample HQ-36, supplementary Table 5). The Main biotite data can thus serve as test to what extent *Perple_X*-computed biotite compositions match the natural record. Such Al^IV^-, Al^VI^- and excess-Al^VI^-contents are shown as function of temperature for P = 7 kbar (experimental pressure in PD93 experiments) as brown, dark-blue and red lines in Fig. 5. Three curves appear for Al^IV^ and Al^VI^, one computed with the biotite activity model Bio(D) from this study, one with Bi(W) and one with Bio(TCC).

With Bio(D), Al^VI^ contents of biotite are predicted that match Main-biotite and experimental PD93 compositions over the whole temperature range of 500 to 900 °C.

Computed Al^IV^- and Al_ex_^VI^– contents agree with PD93 values, but tend to yield overestimated values in the case of Al^IV^, and too low values in the case of Al_ex_^VI^ at low temperatures (Fig. 5). Test calculations show that this could be improved by making some of the *W*’s related to mixing towards *pyp* temperature dependent, assigning them decreasing values with falling temperature.

*Perple_X* computations with Bi(W) yield Al^IV^-contents of biotite in good agreement with natural observations, whereas Al^VI^ is grossly underestimated at T < 700 °C with this model.

*Perple_X* calculations with Bio(TCC), on the other hand give a too large Al^IV^, but a correct Al^VI^–T behaviour with Al^VI^ values close to that obtained using Bio(D).

#### Predicted vs. observed X_Fe_ in biotite

**Fig. 6:**
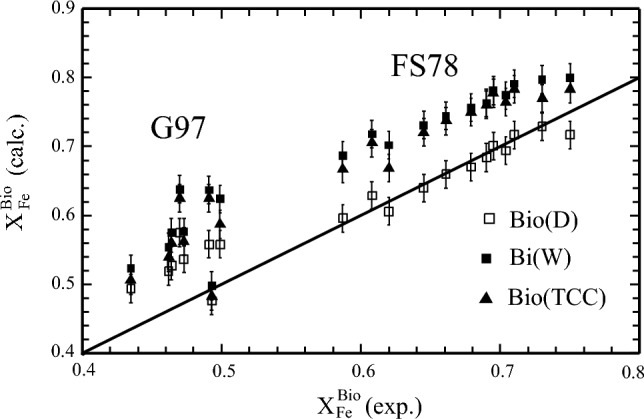
X_Fe_ = Fe/(Fe + Mg) in biotite from Fe–Mg exchange experiments between garnet and biotite of Ferry and Spear (1978 – FS78) and of Gessmann et al. (1997 – G97). Experimentally obtained values (X_Fe_^exp^) are compared to *Perple_X*-calculated ones (X_Fe_^calc^) using the biotite activity model Bio(D) and thermodynamic properties of biotite end-members from this study (open squares), using Bi(W) – filled squares, or using Bio(TCC) – filled triangles. Fe includes Fe^2+^ and Fe^3+^. Bulk compositions are given in supplementary Table 5. Error bars represent ± 2 σ

For bulk compositions as used in the Fe–Mg exchange experiments between garnet and biotite of Ferry and Spear (1978 – FS78) and Gessmann et al. (1997 – G97) (supplementary Table 5), recalculated X_Fe_ in biotite, derived from *Perple_X* calculations using Bio(D) and thermodynamic properties of biotite end-members from this study (Tables 2 and 3), is in good agreement with experimental FS78-X_Fe_ and somewhat larger (by ca. 0.05 mol%) for G97-X_Fe_ (data are compiled in supplementary Table 3, note that X_Fe_ includes both ferrous and ferric iron). This is shown in Fig. 6 (open squares) and was already noted by Dachs and Benisek (2021b).

Similar calculations with Bi(W) and Bio(TCC) yield a mostly ca. 0.1 too large X_Fe_ in biotite in equilibrium with garnet. Bulk X_Fe_ in these experiments was 0.9 (FS78) and 0.78 (G97), respectively.

**Fig. 7:**
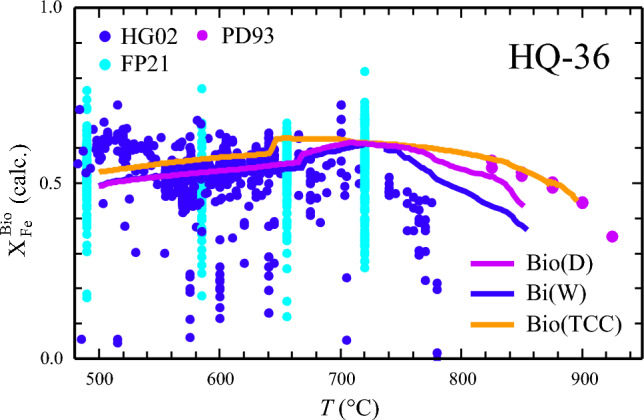
X_Fe_ = Fe/(Fe + Mg) in biotite from the experiments of Patino Douce et al. (1993 – PD93) – data > 800 °C, and from natural sources: dark-blue: Henry and Guidotti (2002 – HG02), Main biotite data set (n = 562), light-blue: Forshaw and Pattison (2021 – FP21), n = 452. *Perple_X*-computed X_Fe_ of biotite using the biotite activity model Bio(D) and thermodynamic properties of biotite end-members from this study is shown as thick line in magenta (P = 7 kbar, bulk-composition HQ-36). For comparison, similar calculations with Bi(W) and Bio(TCC) and thermodynamic dataset values for biotite are also shown (blue and brown thick lines)

For bulk-X_Fe_ around 0.5, a value more typical for common metapelites, all three activity models give a rather similar X_Fe_ vs. T behaviour for biotite at temperature below ~ 800 °C (Fig. 7). For the PD93 experiments, recalculated X_Fe_ is a few mol% lower with Bio(D), to a larger extent lower with Bi(W), and in good agreement with experimental values using Bio(TCC) as activity model (data are compiled in supplementary Table 2).

### Significance of the Castep-computed micro-*w*’s

Our results obtained on Fe–Mg same-site and cross-site interactions (Table 5) indicate that these are weak, confirming that Fe–Mg mixing in biotite is ideal or nearly so, as already suggested in the petrological literature several times since Müller (1972). As demonstrated by BD24, this applies not only for biotite, but for brucite, pyroxene, olivine, spinel, garnet and perovskite as well, suggesting that ideal Fe–Mg mixing could possibly be more the rule than the exception in many solid-solutions. This would have the impact that activity models could be considerably simplified. We tested this for biotite by reevaluating, under the assumption of ideal Fe–Mg mixing (leaving other W’s the same), the experimental partitioning data of Fe and Mg between biotite and olivine of Zhou (1994), as used by Dachs and Benisek (2021a) to extract $\Delta {H}_{f,ann}^{o}$. = -5131.6 ± 2.3 kJ/mol along with weakly asymmetric Fe–Mg non-ideality (Tables 2 and 3). This gives a by 2 kJ different value of $\Delta {H}_{f,ann}^{o}$. = -5133.6 ± 2.5 kJ/mol. The corresponding model is termed ‘Bio(Did)’ and, in addition to Bio(D), Bi(W) and Bio(TCC), was also applied to the garnet/biotite exchange experimental conditions (supplementary Table 3) and to one test sample in the chapter below. Comparing calculated vs. measured biotite compositions, it turns out that the performance of such an ideal Fe–Mg biotite mixing model is almost as good as that of Bio(D).

As emphasized by BD24, there is good agreement between macroscopic *W* of the *phl*-*eas* join computed from reassembling *Castep*-derived micro-*w*’s (18.8 kJ/mol), compared to macroscopic *W*_phleas_ computed independently from applying the single-defect-method to that join (19 ± 3 kJ/mol), as well as resulting from calorimetry (Circone and Navrotsky 1992) and from line-broadening in IR spectra (Dachs and Benisek 2019) (19 – 24.5 kJ/mol). This places some confidence that the calculated micro *w*’s are significant, even when charged cells are used. DFT methods thus seem capable to yield reasonable micro-*w*’s of solid solutions. This opens the possibility towards creating a new generation of activity models, that use DFT- and thus physically based micro-*w*’s and the accordingly reassembled macro-*W*’s for petrological calculations. The biotite activity model of this study – Bio(D) – is a first example in this respect. Due to the large number of required interaction parameters in the model, *Castep* calculations in the present study were, however, confined to the most important binaries involving *phl* and *ann* and some assumptions still were made, e.g. concerning *pyp*- and *tbio*-mixing with the other biotite end-members.

## Test of the new biotite model

Five natural samples of metapelites and metagreywackes, from low- to high grade metamorphic conditions, were chosen to test the new biotite activity model. Their bulk-compositions are given in supplementary Table 5 and are plotted in Figs. 2a and b, showing that they cover SiO_2_-, Al_2_O_3_- and TiO_2_ contents of ~ 52 to 76 wt.%, ~ 11 to 28 wt.% and ca. 0.5 to 1.3 wt.%, respectively. Mineral-chemical data of relevant phases and assemblages, along with published P–T estimates, are compiled in supplementary Table 6. Recalculated mineral compositions using *Perple_X* also appear in this table for each sample, allowing a direct comparison of computed results obtained with Bio(D), Bi(W) and Bio(TCC) with observed biotite compositions (all other activity models were identical). *Perple_X*-generated raw-pseudosections for all samples are given in the Online Resource 7, only that for X567 appears in the text. Note that a Mn end-member has also been added to the Bio(D) model for general application. Similar to Bio(TCC), ideal mixing of all biotite end-members with this Mn-end-member is assumed.

### Low-grade chlorite-biotite zone samples 16 and 18

Mather (1970) studied the biotite isograd in lower greenschist facies metapelites and -greywackes of the Dalradian geological unit in Scotland and provided mineral-chemical, as well as bulk-rock chemical data of these rocks. Metapelite sample 16 is located close to the biotite isograd, still in the chlorite zone, whereas sample 18 is a higher grade SiO_2_-rich metagreywacke from the biotite zone (Mather 1970, their Fig. 1). Both samples contain biotite + chlorite (in major amounts in sample 16, in minor amounts in sample 18) together with phengite, plagioclase, quartz and calcite. As shown by Mather (1970, Table 3), K_D_ = (Fe/Mg)^Chl^/(Fe/Mg)^Bio^ decreases with metamorphic grade. *Perple_X* computed pseudosections using Bio(D) for samples 16 and 18 are shown in Online Resource 7 (Figs. S1a, b) and reveal the observed assemblage biotite-chlorite-phengite-albite-quartz-calcite at low temperatures with the additional phases sphene and epidote in small amounts < 3 vol.%.

**Fig. 8:**
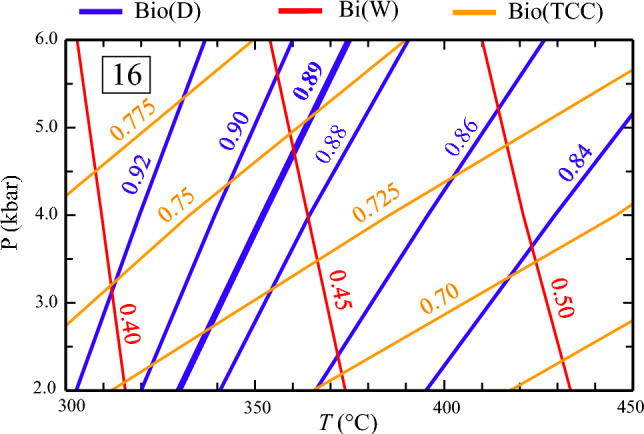
*Perple_X*-generated iso-K_D_ contours for sample 16 (Mather 1970). K_D_ = (Fe/Mg)^Chl^/(Fe/Mg)^Bio^ describes the Fe–Mg distribution between chlorite and biotite, contours in blue were calculated with Bio(D), in red with Bi(W) and in brown with Bio(TCC). Thick blue iso-K_D_ line represents K_D_ = 0.89, as measured in the natural sample

K_D_ as function of P and T is plotted in Fig. 8 for metapelite sample 16, allowing comparison of K_D_-contours calculated with Bio(D), Bi(W) and Bio(TCC). Bio(D)-computed contours indicate decreasing K_D_ with temperature (Fig. 8, blue). This is in agreement with the field observation reported in Mather (1970) and could be taken as an indication that biotite and chlorite in the natural low-grade samples approached equilibrium compositions. The contour matching the observed K_D_ = 0.89, is marked in bold and would indicate a temperature of 350 °C at P = 4 kbar for this sample. On the other hand, K_D_’s that are significantly lower than the observed one for sample 16 result from using Bi(W) and Bio(TCC), respectively.

Mineral-chemical parameters of biotite, chlorite and phengite at 350 °C/4 kbar are given in supplementary Table 6. Bio(D)-calculated Al^VI^ is with 0.26 apfu close to the observed value of 0.31 apfu, while Bi(W)- and Bio(TCC)-predicted Al^VI^-contents are far too low (around 0.05 apfu). All three biotite activity models predict a too low Ti-content (around 0.04 vs. 0.12 apfu). This indicates that the *tbio* end-members in these three models, which only differ in the octahedral site-assignement for Ti, but similarily assume deprotonation of the hydroxyl site to achieve charge balance, a scenario that seems to be more relevant at high metamorphic grades (e.g., Cesare et al. 2003), don’t work so well at low temperatures. Computed X_Fe_^2+^ in biotite and chlorite amount to 0.58 and 0.55 using Bio(D), compared to 0.62 and 0.60 as in sample 16. Due to the comparable X_Fe_^2+^-difference (0.02–0.03), K_D_’s are nevertheless similar. Analogous X_Fe_^2+^-values calculated with Bi(W) and Bio(TCC) are 0.63 and 0.43, and 0.60 and 0.52, respectively, resulting in predicted K_D_’s that are lower than observed.

Similar mineral-chemical features as described above for sample 16, apply for metagreywacke sample 18 concerning its Al^VI^- and Ti content in biotite (supplementary Table 6). The naturally observed K_D_ = (Fe/Mg)^Chl^/(Fe/Mg)^Bio^ in this sample is 0.88 and chlorite vanishes from the biotite-chlorite-phengite-albite-quartz-calcite assemblage above ca. 410 °C at 4 kbar according to the pseudosection (Fig. S1b in Online Resource 7). At these conditions and using Bio(D), computed K_D_ (0.94) somewhat overestimates observed K_D_, whilst with values of 0.48 and 0.75 computed K_D_ is grossly and somewhat underestimated when *Perple_X* calculations are done with Bi(W) and Bio(TCC), respectively.

### Medium-grade andalusite-staurolite zone sample 980A

Sample 980A stems from the staurolite-andalusite zone of the Silurian Waterville Formation in south-central Maine and contains the assemblage garnet-phengite-biotite-staurolite-andalusite-plagioclase-quartz (Ferry 1980). Classical thermobarometry performed by Ferry (1980) revealed pressures around 3.5 kbar for this area and temperatures ranging between 474 and 554 °C for 980A. Tinkham et al. (2001, their Fig. 12) constructed a MnNCKFMASH P–T pseudosection for this sample and discussed the sequence of predicted and observed assemblages for 980A (Tinkham et al. 2001, their Table 2b). A staurolite + andalusite bearing paragenesis does not appear in their pseudosection, but staurolite is confined to a garnet-biotite-staurolite-muscovite assemblage at ca. 4–5.5 kbar. As can be seen from our recalculated MnNCKFMASHTO-pseudosections for 980A (Fig. S2a-c in Online Resource 7), the observed sequence of assemblages, irrespective which activity model is used for biotite, is correctly predicted, i.e. there is a biotite + chlorite-, followed by a biotite + chlorite + garnet-field at low temperatures (P = 3.5 kbar) passing into a staurolite- and finally an andalusite-bearing field with a small zone of stable biotite + garnet + staurolite + andalusite (+ phengite + plagioclase + ilmenite + quartz) in between. The reason for this better match between predicted and observed assemblages compared to Tinkham et al. (2001) is probably due to the inclusion of TiO_2_ in our pseudosection calculation and/or improved thermodynamic data since the work of these authors. One notable difference in the topology of the Bio(D)- compared to the Bi(W)- or Bio(TCC)-calculated pseudosections for sample 980A is that Bio(D) predicts chlorite to react out before staurolite comes in (Fig. S2a in Online Resource 7). It remains to be tested, if this applies for metapelite rock compositions in general.

The mineral chemistry of phases constituting the 980A-assemblage (biotite, staurolite, garnet, plagioclase) is relatively well reproduced by all three biotite activity models (supplementary Table 6). The amount of Al^VI^ in biotite calculated with Bio(D) or Bio(TCC) matches best the observed value, while it’s too low resulting from Bi(W). The Ti-content in biotite from 980A (0.09 apfu) is slightly overestimated with Bio(D) (0.12 apfu) and underestimated with Bi(W) and Bio(TCC) (0.08 and 0.07 apfu). X_Fe_^2+^ in biotite and staurolite, as well as plagioclase and garnet compositions are passably predicted by all three models, with the exception of the spessartine content in garnet, which is generally overestimated. The deviation between observed and calculated X_Fe_^2+^ for biotite and staurolite is 0.04 for Bio(D). Bi(W) and Bio(TCC) predict a twice this value lower X_Fe_^2+^ in staurolite.

### High-grade sample X567

Pitra and de Waal (2001) studied high-grade metapelites from the Marble Hall Fragment of the Bushveld Complex. They inferred two intrusion related metamorphic episodes: (A) an early-stage paragenesis of chiastolitic andalusite-cordierite-biotite-quartz ± garnet equilibrated at 550–600 °C and ca. 2kbar. In sample X567, this early paragenesis transformed to (B) the peak-event assemblage cordierite-biotite-kalifeldspar-quartz ± garnet that constitutes the matrix, whereas a symplectitic intergrowth of cordierite + spinel has replaced the andalusite porphyroblasts. From their phase diagram analysis, based on pseudosections constructed for the simplified KFMASH system, Pitra and de Waal (2001) argue for temperatures of 720–760 °C, conditions of reduced water activity for this second high-grade metamorphic event and a prograde nature for the formation of the cordierite + spinel symplectites.

Similar KFMASH pseudosections for X567 have been calculated by Tajčmanova et al. (2009) and mor recently by Dachs et al. (2021b) using a former version of Bio(D) that was confined to this simplified system.

**Fig. 9:**
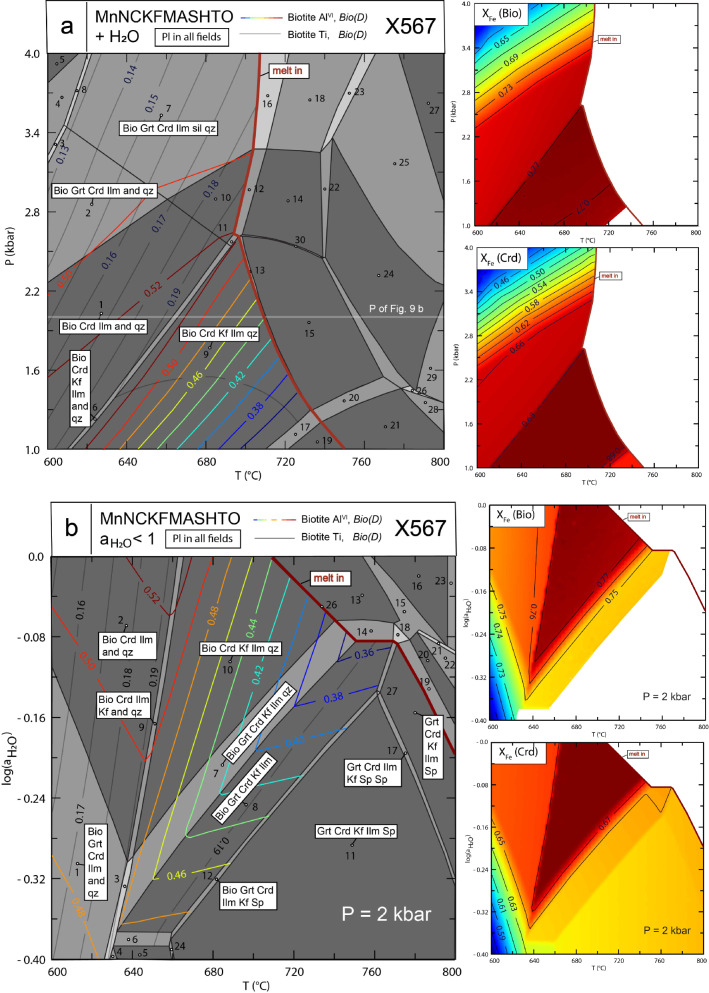
**a)** P–T pseudosection for water-saturated conditions, **b)** log(a_H2O_)-T pseudosection for reduced water-activity conditions at 2 kbar for sample X567 (Pitra and de Waal 2001; bulk-rock composition is given in supplementary Table 5). The observed sequence of parageneses is consistent with the succession of phase-fields at reduced a_H2O_ around 0.7 (i.e., log(a_H2O_) around -0.16). Calculations were done with *Perple_X* using Bio(D) of this study as activity model for biotite (Tables 2 and 3) and imposing logf_O2_-conditions of the QFM buffer. Note that no attempt has been made to compute supra-solidus equilibria in **a)** for a realistic fixed bulk-H_2_O content. Coloured/dark-grey isopleths are the Al^VI^-, respectively the Ti content in biotite, X_Fe_ in biotite and in cordierite are shown in separate colour density plots. Mineral abbreviations for sub-solidus phase-fields are: and andalusite, Bio biotite, Crd cordierite, Grt garnet, Ilm ilmenite, Pl plagioclase, qz quartz, Kf kalifeldspar, sil sillimanite, Sp spinel (field-numbers are explained in Fig. S3 of Online Resource 7)

With the extended Bio(D) model of this study the sequence of parageneses documented for X567 can be consistently reproduced and the predicted biotite composition agrees well with the observed one. This is shown in the pseudosections of Fig. 9, calculated for X567 using Bio(D). Figure 9a is a P–T pseudosection for water-saturated conditions, Fig. 9b is a T-log(a_H2O_) pseudosection for P = 2 kbar. At this pressure and H_2_O in excess, the early-stage (A) paragenesis andalusite-cordierite-biotite-quartz(-plagioclase-ilmenite) ± garnet is stable up to temperatures around 660 °C, grading into the peak-event (B) assemblage cordierite-biotite-kalifeldspar-quartz ± garnet extending up to ca. 710 °C, where the wet solidus is reached. Computed mineral compositions using Bio(D) in this field closely match observed ones at 690 °C/2 kbar (supplementary Table 6). As discussed by Pitra and de Waal (2001), a more realistic scenario for the metamorphic evolution of X567 are, however, water undersaturated conditions (Fig. 9b). The transition from the early-stage to the peak-stage paragenesis is only slightly shifted to lower T’s with decreasing water activity. The observed Al^VI^-content of 0.48 apfu in biotite is correctly predicted, the corresponding isopleth runs through the (B) stability field. The Ti-content remains nearly constant throughout this field and agrees with the measured one (0.19 apfu in X567). For a reduced water activity of a_H2O_ = 0.7 (log(a_H2O_) = -0.16), the peak-paragenesis would indicate ~ 665 °C at P = 2 kbar, based on the Al^VI^-content of 0.48 apfu in biotite. The calculated X_Fe_^2+^  = 0.77 is also close to its measured value of 0.78 (for further mineral-chemical parameters at these conditions, compared to measured ones see supplementary Table 6). Comparable conditions, resulting from the KSMASH system, have been discussed by Dachs et al. (2021b) as ‘best match’ conditions, i.e., where measured and computed compositions come nearest. With the extension of the former KFMASH biotite activity model they used to the present Bio(D) model, this match is still improved (supplementary Table 6).

With rising temperature, cordierite + spinel bearing assemblages, without any melt involvement, become stable (above ca. 750 °C for a_H2O_ = 0.7), confirming Pitra and de Waal (2001)’s opinion that these cordierite + spinel symplectites could have a prograde character.

When pseudosection calculations for X567 are repeated with Bi(W) (Fig. S4 in Online Resource 7), the stability field topology and thus succession of parageneses, as discussed above, remain quite similar, as well as roughly the isopleths slopes. Absolute computed values of the Al^VI^- and Ti contents in biotite are, however, too small to achieve a match to measured analogous quantities in sample X567 (see supplementary Table 6 for comparison of other mineral-chemical parameters). Interestingly, Al^VI^, as predicted for the peak-event (B) assemblage by Pitra and de Waal (2001), using older thermodynamic data and activity models, is in better agreement with observation (their Fig. 5c, showing Al^VI^ -isochores between 0.3 and 0.5).

With the use of Bio(TCC), the stability field of the peak-event (B) assemblage shrinks to pressured < 2 kbar at water-saturated conditions (Fig. S5 in Online Resource 7). At a reduced water activity of a_H2O_ = 0.7 (log(a_H2O_) = −0.16), the Al^VI^-isochore representing Al^VI^ in the natural sample (0.48) would indicate a temperature of ca. 730 °C, around 60 °C higher than resulting from Bio(D). The Ti content and X_Fe_^2+^ in biotite are, however, somewhat larger than measured in sample X567. For the formation of the cordierite + spinel symplectites, temperatures close to 800 °C would be required.

### (Ultra-)High-pressure sample 16Slo12

Li et al. (2020) derived a polymetamorphic P–T history for metapelite 16Slo12 from the (ultra-)high pressure terrane of the Pohorje Mountains in the Eastern Alps (Slovenia) applying various petrological methods, including pseudosection calculations with *Perple_X*, combined with monazite age dating. The P–T conditions of the earliest Permian metamorphic event, succeeded by an Eoalpine and a Tertiary metamorphic episode, were constrained as 7.5 – 10 kbar at 600–650 °C based on the stability field of the inclusion assemblage bio + stau + ru appearing in porphyroblastic garnet of this rock.

We have repeated Li et al. (2020)’s pseudosection calculation using the bulk-rock composition of 16Slo12 (Fig. S6 in Online Resource 7). All three biotite activity models give rather similar stability fields for this inclusion assemblage consistent with that computed by these authors (pink field in their Fig. 10). Calculated mineral compositions are compared in supplementary Table 6 for pressures of 7 and 9 kbar at T = 630 °C. With a value of 0.52 apfu, compared to 0.54 apfu (measured), octahedral Al is best predicted by Bio(D) at 7 kbar, followed by Bio(TCC), whereas Bi(W) yields a low Al^VI^ of 0.27 apfu. Computed X_Fe_^2+^ is generally larger in the order of 0.1 – 0.2 than measured X_Fe_^2+^ in bio of this inclusion assemblage. This discrepancy may be caused, at least partly, by adjustment of the Fe/Mg ratios in biotite, staurolite and garnet to changing P–T conditions during the subsequent Alpine metamorphic (high-pressure) overprints.

## Conclusions

Due to the inclusion of the new biotite components *tbio* and *fbio* to the former KFMASH model of Dachs and Benisek (2021a), the biotite activity model presented in this study (named Bio(D) in *Perple_X*), is more generally applicable, because the Ti- and Fe^3+^ contents of natural biotites can be taken into account. Furthermore, it is the first model that allows the prediction of excess octahedral Al, as present in almost all natural biotites, through incorporation of a *pyp* component.

DFT-based phonon-calculations were applied to derive the heat capacity functions and standard entropies of *tbio* and *fbio* (calorimetric measurements could not be used for that purpose, because it is not possible to synthesize these end-members in a pure form). $\Delta {H}_{f,tbio}^{o}$ was then extracted from experimental data and is in good agreement with its DFT-calculated analogue. The only thermodynamic quantity that was derived from natural data is $\Delta {H}_{f,fbio}^{o}$. Generally we think that the use of estimated P–T data combined with measured mineral-chemistries from the ‘natural lab’ for extracting thermodynamic (mixing) quantities should be kept to a minimum, because such a procedure represents a classical ‘circulus vitiosus’. The derived parameters cannot be expected to have a physical relevance but may merely represent fitting quantities.

As shown recently by Benisek and Dachs (2024) and again demonstrated herein, DFT methods seem capable of quantifying microscopic interaction parameters (micro-*w*’s). For Mg–Al mixing in biotite these are: same-site *w*_MgAl(M1)_ = 82.5 kJ/mol, same-site *w*_SiAl(T1)_ = 95.6 kJ/mol, cross-site ${w}_{MgAlAlSi(M1T1)}=$ 175.1 kJ/mol. The recombination of these micro-*w*’s according to Eq. (10) then gives a macroscopic *W*_phleas_ = 18.8 kJ/mol, as used in the *Perple_X* calculations of this study. Three lines of evidence confirm such a *W*_phleas_ close to 20 kJ/mol and the underlying micro *w*’s: a) results from calorimetry (22.8 ± 18.7 kJ/mol, Circone and Navrotsky 1992), (b) line-broadening in IR spectra of members from the *phl*-*eas* binary (25.4 kJ/mol, Dachs and Benisek 2019), c) independent DFT-calculations applying the single-defect method to the *phl*-*eas* join (19.0 ± 3.0 kJ/mol, Benisek and Dachs 2024). Due to the dependence of *H*_mix_ on the oxygen packing fraction, interaction parameters are not interchangeable between mineral groups, as demonstrated by Benisek and Dachs (2024).

Micro-*w*’s relevant for Mg-Fe mixing in biotite (*w*_MgFe(M1)_, *w*_MgFe(M2),_ ${w}_{MgMgFeFe(M1M2)}$), have only small negative values that are zero within error.

The following general implications can be drawn for the use of Bio(D) in a pseudosection calculation: (i) Bio(D) gives the best match between observed and calculated Al^VI^ compared to Bi(W) or Bio(TCC) (Fig. 5, supplementary Table 6); (ii) X_Fe_^(2+)^ in biotite is most accurately predicted with Bio(D) for Fe-rich bulk-compositions as used in Fe–Mg exchange experiments (Fig. 6), at more intermediate bulk Fe/Mg ratios all three models yield rather similar X_Fe_^2+^, deviating not more than 0.05 mol fractions from measured values in the test samples (supplementary Table 6); (iii) the Ti-contents in biotite is underestimated by all three models in the low-grade test samples. In the higher grade samples a reasonable match is observed for calculations with Bio(D) (deviation ≤ 0.03 apfu), whereas Bi(W) tends to slightly under- and Bio(TCC) to somewhat overestimate measured Ti contents (supplementary Table 6, Fig. 4); (iv) for high_Ti systems, Bio(D), as well as Bi(W) are likely to underestimate Ti-contents in biotite; (v) the suggested published successions of parageneses as determined from petrographic studies of the test samples can be consistently interpreted based on the phase-field topologies resulting from Bio(D). These phase-relations do not differ significantly from that as would be obtained by using Bi(W). With Bio(TCC), some phase-equilibria are shifted to maximal 20 °C higher temperatures or ~ 0.7 kbar lower pressures.

Further extension of the Bio(D) model should consider the introduction of two more end-members, taking into account the Na- and F-content in natural biotites.

The biotite activity model of this study may considered a first example of next-generation activity models that utilize DFT methods combined with experimental phase-equilibrium data to derive unknown thermodynamic standard state properties of end-members and their mixing behaviour and that do not longer resort on heuristic assumptions in parameterizing their interaction parameters. Benisek and Dachs (2024) presented *Castep*-derived micro-*w*’s for the Mg–Al, Si-Al, Mg-Ti, Mg-Ca and Mg-Fe substitutions. By extending their work to a comprehensive set of relevant exchanges and taking the dependence of micro-*w*’s on the type of mineral-group, i.e., on the mineral-specific oxygen packing fraction into account, it should be possible in the future to formulate activity models for petrological use that are based on a consistent set of micro-*w*’s and reassembled macro-*W*’s.

## Supplementary Information

Below is the link to the electronic supplementary material.

Supplementary file1 (PDF 138 KB)

Supplementary file2 (PDF 318 KB)

Supplementary file3 (PDF 202 KB)

Supplementary file4 (PDF 230 KB)

Supplementary file5 (PDF 131 KB)

Supplementary file6 (PDF 193 KB)

Supplementary file7 (PDF 3240 KB)

## Data Availability

All available data are given in the text and in the supplementary information (Online Resources 1–7).
