# Supplementary material for: A new activity model for biotite and its application
Source: Contrib Mineral Petrol. 2024 Sep 30;179(10):93. doi: 10.1007/s00410-024-02173-6 (PMC11452188; doi:10.1007/s00410-024-02173-6)
Supplement: Supplementary file 1 — Supplementary file1 (PDF 138 KB) [file 410_2024_2173_MOESM1_ESM.pdf]

Edgar Dachs and Artur Benisek (2024): "A new activity model for biotite and its application"
(Contributions to Mineralogy and Petrology, in press)
Department of Chemistry and Physics of Materials, University of Salzburg
Jakob-Haringerstrasse 2a, A-5020 Salzburg, Austria
E-mail: edgar.dachs@plus.ac.at

Supplementary Table 1a Thermodynamic data of *phl, ann, east, tbio* and f*bio* to be inserted into *Perple_X* thermodynamic data file <hp62ver.dat>

```
phlD      EoS = 8 | H=  -6209867.  from Dachs and Benisek (2019), required for Bio(D)
MgO(3)Al2O3(.5)SiO2(3)K2O(.5)H2O(1)
GH = -6308534  S0 = 330.93  V0 = 14.964
c1 = 920.883  c2 = -.8451119E-1  c3 = 2105320  c5 = -9802.57
b1 = .38E-4  b5 = 500.325  b6 = 513000  b7 = -.143E-4  b8 = 7.33
end


annD      EoS = 8 | H=  -5131546  from Dachs and Benisek (2021), required for Bio(D)
Al2O3(.5)SiO2(3)K2O(.5)FeO(3)H2O(1)
GH = -5257643  S0 = 422.93  V0 = 15.48
c1 = 748.96  c2 = -.0581675E-1  c3 = -921630  c5 = -6095.58
b1 = .38E-4  b5 = 416.5931  b6 = 513000  b7 = -.143E-4  b8 = 7.33
end


annDid     EoS = 8 | H=  -5133636.5  from Dachs and Benisek (2024), required for Bio(Did), ideal Fe-Mg mixing
Al2O3(.5)SiO2(3)K2O(.5)FeO(3)H2O(1)
GH = -5259733  S0 = 422.93  V0 = 15.48
c1 = 748.96  c2 = -.0581675E-1  c3 = -921630  c5 = -6095.58
b1 = .38E-4  b5 = 416.5931  b6 = 513000  b7 = -.143E-4  b8 = 7.33
end


eastD     EoS = 8 | H=  -6352012 from Dachs and Benisek (2019), required for Bio(D)
MgO(2)Al2O3(1.5)SiO2(2)K2O(.5)H2O(1)
GH = -6439816  S0 = 294.5  V0 = 14.65
c1 = 852.5  c2 = -.586602E-1  c3 = -404300  c5 = -8426.84
b1 = .38E-4  b5 = 509.0324  b6 = 530000  b7 = -.143E-4  b8 = 7.33
end


tbioD      EoS = 8 | tbioD: H=  -6131113 from Dachs and Benisek (2024), required for Bio(D)
Al2O3(.5)SiO2(3)K2O(.5)MgO(2)TiO2(1)
GH = -6228924  S0 = 328.06  V0 = 14.808
c1 = 798.37  c2 = -.597056E-1  c3 = 58239.6  c5 = -7792.34
b1 = .38E-4  b5 = 416.5931  b6 = 632800  b7 = -.143E-4  b8 = 9.08
end


fbioD      EoS = 8 | H=  -5933628   from Dachs and Benisek (2024), required for Bio(D)
Al2O3(1)SiO2(2)K2O(.5)MgO(2)FeO(1)H2O(1)O2(.25)
GH = -6023579  S0 = 301.693  V0 = 15.414
c1 = 777.684  c2 = -.396494E-1  c3 = -2838850  c5 = -6422.47
b1 = .38E-4  b5 = 416.5931  b6 = 513000  b7 = -.143E-4  b8 = 7.33
end
```

Supplementary Table Table 1b Solution model Bio(D) to be inserted into *Perple_X* data file <solution_model.dat>

```
                  --------------------------------------------------------
begin_model
                  Fe-Mg-Al(Ts)-Al(ex)-Ti-Fe3+-Mn Biotite: Dachs and Benisek (2024)

                  688 format, E. Dachs  Jun/2024.

                  Polytope 1 = [V][V][T']
                  Polytope 2 = [K][M,T][M]

                  where

                  T = Al on M1, T' = Al on M2
                  M = Mg, Fe


                                  1      2      3      4      H
                                  A      M1     M2     T1
                                  ______________________________________________

                       Mutliplicity   1      1      2      2      2
                                  ______________________________________________

                       endmember                                     type
                       _________                                     ________

             polytope 1:   1: pyp      V      V      AlAl    SiSi    OH   independent orphan
                              mnbi     K      Mn     MnMn    AlSi    OH

             polytope 2:   2: ffbio    K      Fe3    FeFe    AlAl    OH   dependent
                           3: fbio     K      Fe3    MgMg    AlAl    OH
                           4: sid      K      Al     FeFe    AlAl    OH   dependent
                           5: east     K      Al     MgMg    AlAl    OH
                           6: ftbio    K      Fe     FeTi    AlSi    O    dependent
                           7: tbio     K      Mg     MgTi    AlSi    O
                           8: ann      K      Fe     FeFe    AlSi    OH
                           9: phl      K      Mg     MgMg    AlSi    OH

          ordered species: 10: obi     K      Fe     MgMg    AlSi    OH   ordered


Bio(D)                   | model name
abbreviation Bio
full_name    biotite

688                      | model type: 688 format standard model

2                        | number of polytopes
                         | polytope names and composite composition space subdivision schemes
[V][V][T']    0   0.5   .1   0    |
[K][M,T][M]   by difference
                         | ---------------------------
                         | Polytope 1 - 1 simplex
1                        | number of simplices
2   1                    | number of vertices on each simplex
prl      mnbi            | endmembers on the vertices

X_Mn    0    0.5   .1  0       | X(1,1) is bulk Fe/M
X_Prl  by difference
```

```
                        | ---------------------------
                        | Polytope 2 - 2x4 simplices
2                       | number of simplices
2  4                    | number of vertices on each simplex
                        | endmembers on the vertices
ffbio    fbioD
sid      eastD
ftbio    tbioD
annD     phlD
                        | First 2-simplex
X_Mg    0    1   .1  0           | X(1,1) is bulk Fe/M
X_Fe    by difference
                        | Second 2-simplex
X_FeTs  0    1   .1  0           | X(2,1) is Fe3/[T+M] on M1, M on M2
X_AlTs  0    1   .1  0           | X(2,2) is Al/[T+M]  on M1, M on M2
X_TiTs  0    1   .1  0           | X(2,3) is MTi/[M+MTi] on M2, M on M1
X_MBio  by difference

begin_ordered_endmembers
obi  = 2/3 phlD + 1/3 annD    enthalpy_of_ordering = -2.0d3
end_ordered_endmembers

begin_dependent_endmembers
sid = 1 annD + 1 eastD - 1 obi
ftbio   = 1 tbioD + 1/2 annD  + 1/2 obi - 1 phlD
ffbio   = 1 fbioD + 1 annD    - 1 obi
end_dependent_endmembers


begin_excess_function
W(phlD annD annD)    -8800.   | excess parameters from Dachs and Benisek (2021, 2024)
W(phlD phlD annD)    14300.
W(phlD eastD)  19000.
W(phlD obi)     -100.
W(phlD prl)  116800
W(annD eastD)  -5000.
W(annD obi)    -400.
W(annD prl)  108200
W(annD tbioD) -30000.
W(eastD obi)  -5000.
W(eastD prl) 120000
W(obi prl)  120000
W(prl tbioD) 120000
W(prl fbioD) 120000
end_excess_function



5                         | number of identisites in configurational entropy model
A
2 1 1
z(K,A) = 1 phlD + 1 eastD + 1 annD + 1 obi + 1 tbioD + 1 fbioD + 1 mnbi
z(Vac,A) = 1 prl

M1                          | site name
6 1 1                       | number of species, effective multiplicity, true multiplicity
z(Al,M1)   = 1 eastD
z(Mg,M1)   = 1 phlD + 1 tbioD
z(Fe3,M1) = 1 fbioD
z(Mn,M1)   = 1 mnbi
z(Fe,M1)   = 1 annD + 1 obi
z(Vac,M1)  = 1 prl
```

```
M2                          | site name
5 2 2                       | number of species, effective multiplicity, true multiplicity
z(Fe,M2)   = 1 annD
z(Al,M2)   = 1 prl
z(Ti,M2)   = 1/2 tbioD
z(Mn,M2)   = 1 mnbi
z(Mg,M2)   = 1 eastD + 1 obi + 1 phlD + 1/2 tbioD + 1 fbioD

T1                          | site name
2 2 2                       | number of species, effective multiplicity, true multiplicity
z(Si,T1)   = 1/2 phlD + 1/2 annD + 1/2 obi + 1 prl + 1/2 tbioD + 1/2 mnbi
z(Al,T1)   = 1 eastD + 1/2 phlD + 1/2 annD + 1/2 obi + 1/2 tbioD + 1 fbioD + 1/2 mnbi

OH                          | site name
2 2 2                       | 2 species on H, 2 site per formula unit.
z(O,OH)    = 1 tbioD
z(OH,OH)   = 1 eastD + 1 phlD + 1 annD + 1 obi + 1 prl + 1 fbioD + 1 mnbi

[Si2O10(OH)2]                | formula suffix, enter "none" for no suffix.

end_of_model
```

Supplementary Table 1c Solution model Bio(Did) (ideal Fe-Mg mixing) to be inserted into *Perple_X* data file <solution_model.dat>

```
              --------------------------------------------------------
begin_model
              Fe-Mg-Al(Ts)-Al(ex)-Ti-Fe3+-Mn Biotite: Dachs and Benisek (2024)
              simplified version of Bio(D) with ideal Fe-Mg mixing.

              688 format, E. Dachs  Jun/2024.

              Polytope 1 = [V][V][T']
              Polytope 2 = [K][M,T][M]

              where

              T = Al on M1, T' = Al on M2
              M = Mg, Fe


                          1      2      3      4      H
                          A      M1     M2     T1
                          ________________________________________

                  Mutliplicity   1      1      2      2      2
                          ________________________________________

                  endmember                          type
                  _________                          ________

        polytope 1:   1: pyp     V      V     AlAl    SiSi    OH   independent orphan
                         mnbi    K      Mn    MnMn    AlSi    OH

        polytope 2:   2: ffbio   K      Fe3   FeFe    AlAl    OH   dependent
                      3: fbio    K      Fe3   MgMg    AlAl    OH
                      4: sid     K      Al    FeFe    AlAl    OH   dependent
                      5: east    K      Al    MgMg    AlAl    OH
                      6: ftbio   K      Fe    FeTi    AlSi    O    dependent
```

```
                                      7: tbio         K       Mg       MgTi    AlSi      O
                                      8: ann          K       Fe       FeFe     AlSi     OH
                                      9: phl          K       Mg       MgMg    AlSi     OH

                ordered species: 10: obi          K       Fe       MgMg     AlSi     OH    ordered


Bio(Did)                     | model name
abbreviation Bio
full_name    biotite

688                          | model type: 688 format standard model

2                            | number of polytopes
                             | polytope names and composite composition space subdivision schemes
[V][V][T']    0   0.5   .1   0     |
[K][M,T][M]   by difference
                             |
                             | Polytope 1 - 1 simplex
1                            | number of simplices
2   1                        | number of vertices on each simplex
prl      mnbi               | endmembers on the vertices

X_Mn    0    0.5   .1  0          | X(1,1) is bulk Fe/M
X_Prl  by difference
                             | ----------------------------
                             | Polytope 2 - 2x4 simplices
2                            | number of simplices
2  4                         | number of vertices on each simplex
                             | endmembers on the vertices
ffbio    fbioD
sid      eastD
ftbio    tbioD
annDid   phlD
                             | First 2-simplex
X_Mg    0    1   .1  0            | X(1,1) is bulk Fe/M
X_Fe    by difference
                             | Second 2-simplex
X_FeTs  0    1   .1  0            | X(2,1) is Fe3/[T+M] on M1, M on M2
X_AlTs  0    1   .1  0            | X(2,2) is Al/[T+M]  on M1, M on M2
X_TiTs  0    1   .1  0            | X(2,3) is MTi/[M+MTi] on M2, M on M1
X_MBio  by difference

begin_ordered_endmembers
obi  = 2/3 phlD + 1/3 annDid    enthalpy_of_ordering = -2.0d3
end_ordered_endmembers

begin_dependent_endmembers
sid = 1 annDid + 1 eastD - 1 obi
ftbio   = 1 tbioD + 1/2 annDid  + 1/2 obi - 1 phlD
ffbio   = 1 fbioD + 1 annDid    - 1 obi
end_dependent_endmembers


begin_excess_function
W(phlD eastD)  19000.            | excess parameters from Dachs and Benisek (2021, 2024)
W(phlD prl)  116800
W(annDid eastD)  -5000.
W(annDid prl)  108200
W(annDid tbioD) -30000.
W(eastD obi)  -5000.
W(eastD prl) 120000
```

```
W(eastD tbioD) 0.
W(obi prl)  120000
W(prl tbioD) 120000
W(prl fbioD) 120000
end_excess_function


5                               | number of identisites in configurational entropy model
A
2 1 1
z(K,A) = 1 phlD + 1 eastD + 1 annDid + 1 obi + 1 tbioD + 1 fbioD + 1 mnbi
z(Vac,A) = 1 prl

M1                              | site name
6 1 1                           | number of species, effective multiplicity, true multiplicity
z(Al,M1)   = 1 eastD
z(Mg,M1)   = 1 phlD + 1 tbioD
z(Fe3,M1)  = 1 fbioD
z(Mn,M1)   = 1 mnbi
z(Fe,M1)   = 1 annDid + 1 obi
z(Vac,M1)  = 1 prl

M2                              | site name
5 2 2                           | number of species, effective multiplicity, true multiplicity
z(Fe,M2)   = 1 annDid
z(Al,M2)   = 1 prl
z(Ti,M2)   = 1/2 tbioD
z(Mn,M2)   = 1 mnbi
z(Mg,M2)   = 1 eastD + 1 obi + 1 phlD + 1/2 tbioD + 1 fbioD

T1                              | site name
2 2 2                           | number of species, effective multiplicity, true multiplicity
z(Si,T1)   = 1/2 phlD + 1/2 annDid + 1/2 obi + 1 prl + 1/2 tbioD + 1/2 mnbi
z(Al,T1)   = 1 eastD + 1/2 phlD + 1/2 annDid + 1/2 obi + 1/2 tbioD + 1 fbioD + 1/2 mnbi

OH                              | site name
2 2 2                           | 2 species on H, 2 site per formula unit.
z(O,OH)     = 1 tbioD
z(OH,OH)    = 1 eastD + 1 phlD + 1 annDid + 1 obi + 1 prl + 1 fbioD + 1 mnbi

[Si2O10(OH)2]                   | formula suffix, enter "none" for no suffix.

end_of_model
```
