# Supplementary material for: A new activity model for biotite and its application
Source: Contrib Mineral Petrol. 2024 Sep 30;179(10):93. doi: 10.1007/s00410-024-02173-6 (PMC11452188; doi:10.1007/s00410-024-02173-6)
Supplement: Supplementary file 2 — Supplementary file2 (PDF 318 KB) [file 410_2024_2173_MOESM2_ESM.pdf]

Edgar Dachs and Artur Benisek (2024): "A new activity model for biotite and its application"
(Contributions to Mineralogy and Petrology, in press)
Department of Chemistry and Physics of Materials, University of Salzburg
Jakob-Haringerstrasse 2a, A-5020 Salzburg, Austria
E-mail: edgar.dachs@plus.ac.at

Supplementary Table 2 Experimental data of Patino Douce et al. (1993 - **PD93**), used to extract enthalpy of formation values for the Ti-biotite end-member *tbio* of this study (see text and Tab. 3).
The experimental data of Vielzeuf and Montel (1994) and Patino-Douce and Bird (1995), as well as the natural data of Williams and Grambling (1990) and Holdaway et al. (1997) are also listed.
For reasons discussed in the text, these are only used in the plots of Figs. 1, 4 and 5, but not for extraction purposes.
Perple X-calculated biotite, garnet and ilmenite compositions are given for comparison, applying the biotite activity models Bio(D) and its ideal Fe-Mg mixing version Bio(Did) of this study, and models Bi(W) and Bio(TCC), published by White et al. (2014) and Tajcmanova et al. (2009) for biotite, Gt(W) for garnet (White et al. 2014) and Ilm(WPH) for ilmenite (White et al. 2000) .
Bulk-compositions used in Perplex calculations are given in supplementary Table 5 of this study. fO2-conditions of the QFM-buffer were simulated in the Perplex calculations.

Experimental data of Patino Douce et al. (1993): Ilmenite-bearing experiments (Tab. 1), used for extracting H^{o}_{f}(tbio) of this study.

run	P	T		biotite: experimental and computed composition: apfu (atoms per formula unit - 11 oxygens)										garnet apfu (12 oxygens)					Ilmenite apfu (3 oxygens)				
	kbar	°C	exp. data / biotite activity model	Si	Al (IV)	Al (VI)	Al (VI-ex)	Ti	Fe^{2+}	Fe^{3+}	Mg	K	X_{Fe}^{2+}	X(Py)	X(Alm)	X(Gro)	X(Sps)	X(Kho)	Ti	Mg	Mn	Fe^{2+}	Fe^{3+}
APD-25	7	825	**PD93**	2.656	1.344	0.417	0.073	0.202	1.122	0.068	0.943	0.888	0.543	0.198	0.705	0.034	0.027	0.037	0.987	0.044	0.008	0.938	0.014
			Bio(D)	2.640	1.360	0.466	0.083	0.216	1.154	0.018	1.101	0.959	0.512	0.317	0.623	0.030	0.017	0.013	0.961	0.099	0.006	0.855	0.079
			Bio(Did)	2.627	1.373	0.481	0.083	0.244	1.183	0.016	1.032	0.944	0.534	0.276	0.652	0.038	0.021	0.014	0.959	0.082	0.008	0.813	0.082
			Bi(W)	2.692	1.308	0.264		0.223	1.038	0.045	1.428	1.000	0.421	0.293	0.647	0.030	0.017	0.013	0.959	0.085	0.006	0.855	0.079
			Bio(TCC)	2.480	1.520	0.484		0.291	1.229	0.036	0.956	1.000	0.562	0.206	0.706	0.043	0.031	0.014	not stable				
APD-22	7	850	**PD93**	2.690	1.310	0.352	0.042	0.222	1.153	0.000	1.064	0.877	0.520	0.213	0.720	0.028	0.027	0.012	0.973	0.060	0.005	0.909	0.040
			Bio(D)	2.676	1.324	0.431	0.082	0.237	0.976	0.016	1.297	0.959	0.430	0.361	0.591	0.021	0.014	0.013	0.956	0.114	0.006	0.837	0.077
			Bio(Did)	2.649	1.351	0.460	0.083	0.250	1.094	0.016	1.137	0.948	0.490	0.337	0.610	0.024	0.013	0.013	0.960	0.105	0.006	0.849	0.080
			Bi(W)	2.705	1.295	0.255		0.250	0.896	0.041	1.557	1.000	0.365	0.352	0.599	0.022	0.014	0.013	0.961	0.110	0.006	0.845	0.079
			Bio(TCC)	2.482	1.518	0.483		0.320	1.148	0.035	1.011	1.000	0.532	0.237	0.684	0.040	0.024	0.015	0.955	0.071	0.009	0.875	0.091
APD-33	7	875	**PD93**	2.693	1.307	0.325	0.018	0.254	1.048	0.035	1.103	0.877	0.487	0.261	0.688	0.016	0.010	0.025	0.973	0.072	0.002	0.900	0.040
			Bio(TCC)	2.485	1.515	0.480		0.320	1.111	0.035	1.047	1.000	0.515	0.307	0.635	0.027	0.018	0.015	0.957	0.105	0.007	0.845	0.086
APD-34	7	900	**PD93**	2.673	1.327	0.296	-0.031	0.256	0.963	0.059	1.196	0.897	0.446	0.292	0.647	0.014	0.008	0.039	0.971	0.087	0.002	0.884	0.041
			Bio(TCC)	2.497	1.503	0.470	0.000	0.353	0.924	0.032	1.219	1.000	0.431	0.355	0.598	0.020	0.014	0.015	0.960	0.120	0.006	0.834	0.086
APD-35	7	925	**PD93**	2.661	1.339	0.282	-0.057	0.234	0.775	0.047	1.445	0.892	0.349	0.375	0.581	0.007	0.003	0.035	0.974	0.139	0.002	0.835	0.030
APD-43	7	950	**PD93**	2.646	1.354	0.199	-0.155	0.346	0.740	0.052	1.388	0.909	0.348	0.409	0.532	0.010	0.007	0.042	0.975	0.121	0.001	0.854	0.028
APD-12	10	825	**PD93**	2.686	1.314	0.492	0.178	0.183	1.172	0.010	0.901	0.884	0.565	0.199	0.722	0.037	0.028	0.014	0.971	0.044	0.007	0.922	0.046
APD-11	10	850	**PD93**	2.718	1.282	0.463	0.181	0.208	1.096	0.009	0.964	0.873	0.532	0.201	0.721	0.041	0.025	0.013	0.975	0.055	0.003	0.915	0.041
APD-13	10	875	**PD93**	2.679	1.321	0.401	0.080	0.249	1.037	0.026	1.021	0.882	0.504	0.252	0.697	0.021	0.011	0.019	0.967	0.067	0.003	0.896	0.055
APD-4	10	900	**PD93**	2.690	1.310	0.393	0.083	0.255	0.909	0.031	1.143	0.882	0.443	0.272	0.666	0.024	0.017	0.020	0.961	0.094	0.001	0.871	0.051

Experimental data of Vielzeuf and Montel (1994), analyses in Montel and Vielzeuf (1997):

run	P kbar	T °C	exp. data / biotite activity model	Si	Al (IV)	Al (VI)	Al (VI-ex)	Ti	Fe^{2+}	Fe^{3+}	Mg	K	X_{Fe}^{2+}	X(Py)	X(Alm)	X(Gro)	X(Sps)	X(Kho)	Ti	Mg	Mn	Fe^{2+}	Fe^{3+}
A117C	5	809		2.976	1.024	0.446	0.422	0.189	0.831	0.000	1.166			0.299	0.593	0.040	0.068		1.000	0.000	0.000	1.000	0.000
A117B	5	867		2.913	1.087	0.370	0.283	0.267	0.818	0.000	1.176			0.394	0.523	0.045	0.038		1.000	0.000	0.000	1.000	0.000
A118B	8	864		2.830	1.170	0.265	0.095	0.276	0.647	0.000	1.498			0.364	0.606	0.014	0.016		1.000	0.000	0.000	1.000	0.000
A115C	8	875		2.867	1.133	0.347	0.214	0.206	0.760	0.000	1.402			0.401	0.512	0.067	0.020		1.000	0.000	0.000	1.000	0.000
A118A	8	913		2.826	1.174	0.311	0.137	0.269	0.630	0.000	1.463			0.397	0.570	0.021	0.013		1.000	0.000	0.000	1.000	0.000
A115B	8	919		2.803	1.197	0.248	0.051	0.270	0.751	0.000	1.467			0.464	0.475	0.033	0.028		1.000	0.000	0.000	1.000	0.000
A114B	10	885		2.816	1.184	0.232	0.048	0.267	0.875	0.000	1.358			0.361	0.550	0.077	0.012		1.000	0.000	0.000	1.000	0.000

Experimental data of Patino-Douce and Bird (1995)

run	P kbar	T °C	exp. data / biotite activity model	Si	Al (IV)	Al (VI)	Al (VI-ex)	Ti	Fe^{2+}	Fe^{3+}	Mg	K	X_{Fe}^{2+}	X(Py)	X(Alm)	X(Gro)	X(Sps)	X(Kho)	Ti	Mg	Mn	Fe^{2+}	Fe^{3+}
APD-588	12.5	930		2.777	1.223	0.135		0.371	0.902	0.000	1.299			0.258	0.512	0.194	0.036		0.928	0.000	0.000	0.775	0.000
APD-589	12.5	960		2.747	1.253	0.094		0.411	0.803	0.000	1.387			0.291	0.509	0.181	0.018		0.928	0.000	0.000	0.775	0.000
APD-531	15	925		2.734	1.266	0.155		0.339	1.064	0.000	1.188			0.255	0.531	0.168	0.046		0.973	0.000	0.000	0.873	0.000
APD-547	15	950		2.808	1.192	0.176		0.372	0.795	0.000	1.330			0.269	0.507	0.198	0.026		0.973	0.000	0.000	0.873	0.000
APD-570	15	950		2.807	1.193	0.200		0.375	0.831	0.000	1.254			0.293	0.474	0.205	0.028		0.973	0.000	0.000	0.873	0.000

Natural data of Williams and Grambling (1990)

run	P kbar	T °C	exp. data / biotite activity model	Si	Al (IV)	Al (VI)	Al (VI-ex)	Ti	Fe^{2+}	Fe^{3+}	Mg	K	X_{Fe}^{2+}	X(Py)	X(Alm)	X(Gro)	X(Sps)	X(Kho)	Ti	Mg	Mn	Fe^{2+}	Fe^{3+}
77-85B	3.8	518		2.624	1.376	0.461	0.085	0.091	1.331	0.181	0.762			0.070	0.894	0.033	0.003		1.000	0.000	0.000	1.000	0.000
76-420	3.8	502		2.633	1.367	0.461	0.094	0.090	1.197	0.179	0.889			0.086	0.886	0.025	0.003		0.980	0.000	0.010	0.970	0.040
77-81E	3.8	527		2.627	1.373	0.457	0.084	0.091	1.273	0.190	0.810			0.077	0.876	0.034	0.013		1.000	0.000	0.070	0.930	0.080
77-41	3.8	482		2.676	1.324	0.473	0.149	0.078	1.175	0.176	0.934			0.077	0.850	0.053	0.020		0.970	0.000	0.000	0.970	0.060
78-22A	3.8	504		2.606	1.394	0.468	0.074	0.086	1.406	0.191	0.680			0.053	0.857	0.010	0.080		0.980	0.000	0.020	0.960	0.040
77-73	3.8	493		2.634	1.366	0.422	0.056	0.092	1.186	0.193	0.995			0.081	0.814	0.025	0.080		1.000	0.000	0.040	0.980	0.000
77-23	3.8	484		2.603	1.397	0.458	0.061	0.098	1.438	0.273	0.586			0.038	0.842	0.033	0.087		0.990	0.000	0.130	0.860	0.120
83-45	3.8	487		2.656	1.344	0.456	0.112	0.091	1.228	0.200	0.841			0.062	0.802	0.032	0.104		1.000	0.000	0.040	0.980	0.000
77-46D	3.8	521		2.687	1.313	0.390	0.077	0.076	0.819	0.192	1.344			0.117	0.634	0.052	0.197		0.820	0.000	0.030	0.790	0.360
76-437	3.8	523		2.657	1.343	0.404	0.061	0.105	1.231	0.168	0.914			0.058	0.685	0.043	0.214		1.000	0.000	0.120	0.880	0.000
80-46	4.5	541		2.610	1.390	0.430	0.040	0.110	1.290	0.190	0.780			0.081	0.893	0.016	0.010		0.984	0.000	0.001	0.983	0.032
80-46b	4.5	551		2.630	1.370	0.470	0.100	0.120	1.300	0.180	0.760			0.080	0.894	0.016	0.010		0.991	0.000	0.005	0.986	0.018
80-30	4.5	563		2.570	1.430	0.410	0.000	0.130	1.270	0.190	0.830			0.091	0.876	0.021	0.012		1.000	0.000	0.040	0.960	0.000
80-187	4.5	522		2.650	1.350	0.460	0.110	0.100	1.150	0.170	0.930			0.093	0.857	0.038	0.012		1.000	0.000	0.017	0.933	0.000
80-20	4.5	581		2.600	1.400	0.420	0.020	0.120	1.390	0.210	0.650			0.069	0.896	0.016	0.019		0.972	0.000	0.005	0.967	0.056
81-225	4.5	554		2.720	1.280	0.300	0.020	0.070	0.730	0.140	1.690			0.218	0.678	0.066	0.038		0.891	0.000	0.218	0.883	0.000
80-1922	4.5	525		2.670	1.330	0.450	0.120	0.090	1.070	0.190	1.010			0.093	0.791	0.074	0.042		1.000	0.000	0.031	0.969	0.000
81-234	4.5	585		2.740	1.260	0.360	0.100	0.080	0.810	0.150	1.430			0.183	0.687	0.062	0.068		0.877	0.000	0.018	0.859	0.246
C81-148	4.5	584		2.620	1.380	0.410	0.030	0.090	1.160	0.240	0.920			0.091	0.767	0.051	0.091		0.927	0.000	0.012	0.915	0.000
C81-105	4.5	564		2.640	1.360	0.350	0.000	0.140	1.180	0.210	0.930			0.074	0.722	0.083	0.121		0.934	0.000	0.071	0.863	0.134
W83-302	5	634		2.665	1.335	0.357	0.022	0.089	1.020	0.194	1.175			0.165	0.773	0.049	0.013		0.940	0.000	0.010	0.930	0.120
W84-86	5	530		2.725	1.275	0.343	0.068	0.067	0.697	0.163	1.598			0.169	0.635	0.091	0.105		0.890	0.000	0.020	0.870	0.220
W84-94	5	489		2.664	1.336	0.366	0.030	0.089	1.144	0.202	1.012			0.067	0.740	0.068	0.125		0.910	0.000	0.020	0.890	0.180
W84-130	5	525		2.671	1.329	0.371	0.042	0.096	0.982	0.187	1.173			0.092	0.678	0.064	0.166		0.850	0.000	0.010	0.840	0.280
W84-145	5	503		2.688	1.312	0.382	0.070	0.076	0.964	0.184	1.208			0.087	0.661	0.044	0.208		0.900	0.000	0.010	0.890	0.200

Natural data of Holdaway et al. (1997)

run	P kbar	T °C	exp. data / biotite activity model	Si	Al (IV)	Al (VI)	Al (VI-ex)	Ti	Fe^{2+}	Fe^{3+}	Mg	K	X_{Fe}^{2+}	X(Py)	X(Alm)	X(Gro)	X(Sps)	X(Kho)	Ti	Mg	Mn	Fe^{2+}	Fe^{3+}
27	4	584				0.499		0.100	1.383	0.166	0.682			0.065	0.866	0.024	0.044		1.000	0.000	0.000	1.000	0.000
D084x	4	568				0.463		0.086	1.320	0.159	0.813			0.076	0.846	0.021	0.057		1.000	0.000	0.000	1.000	0.000
D084y	4	566				0.489		0.088	1.298	0.156	0.818			0.077	0.854	0.021	0.048		1.000	0.000	0.000	1.000	0.000
84	4	555				0.492		0.082	1.173	0.141	0.965			0.067	0.677	0.033	0.223		1.000	0.000	0.000	1.000	0.000
D123x	4	572				0.448		0.124	1.146	0.138	0.964			0.085	0.744	0.042	0.128		1.000	0.000	0.000	1.000	0.000
D123y	4	570				0.432		0.104	1.155	0.139	1.033			0.089	0.748	0.042	0.121		1.000	0.000	0.000	1.000	0.000
65-4	4	583				0.483		0.091	1.240	0.122	1.035			0.087	0.739	0.073	0.101		1.000	0.000	0.000	1.000	0.000
114	4	579				0.512		0.079	1.162	0.140	1.001			0.096	0.804	0.064	0.036		1.000	0.000	0.000	1.000	0.000
128	4	567				0.500		0.085	1.156	0.143	1.020			0.083	0.746	0.059	0.112		1.000	0.000	0.000	1.000	0.000
42-2	4	590				0.430		0.094	1.160	0.114	1.026			0.105	0.756	0.033	0.106		1.000	0.000	0.000	1.000	0.000
93	4	593				0.496		0.081	1.024	0.195	1.032			0.103	0.723	0.057	0.118		1.000	0.000	0.000	1.000	0.000
547 A	4	548				0.412		0.079	1.081	0.161	1.099			0.081	0.733	0.056	0.131		1.000	0.000	0.000	1.000	0.000
544 B	4	559				0.504		0.075	1.060	0.128	1.065			0.085	0.724	0.062	0.129		1.000	0.000	0.000	1.000	0.000
D124	4	568				0.487		0.098	1.058	0.128	1.084			0.096	0.725	0.037	0.142		1.000	0.000	0.000	1.000	0.000
49	4	565				0.496		0.086	1.063	0.132	1.103			0.097	0.742	0.040	0.121		1.000	0.000	0.000	1.000	0.000
D233	4	563				0.461		0.077	1.044	0.125	1.157			0.102	0.743	0.045	0.110		1.000	0.000	0.000	1.000	0.000
Ra-a48-66	4	569				0.472		0.078	1.065	0.151	1.103			0.098	0.758	0.062	0.082		1.000	0.000	0.000	1.000	0.000
Ra-a65-66	4	579				0.497		0.086	1.145	0.127	1.037			0.085	0.709	0.078	0.128		1.000	0.000	0.000	1.000	0.000
Ra-c1-66	4	579				0.531		0.085	1.047	0.185	1.087			0.105	0.752	0.038	0.104		1.000	0.000	0.000	1.000	0.000
Ra-c4-66	4	579				0.487		0.084	1.136	0.155	1.056			0.094	0.731	0.044	0.131		1.000	0.000	0.000	1.000	0.000
Ra-a14-66	4	570				0.457		0.084	1.050	0.130	1.081			0.094	0.722	0.058	0.126		1.000	0.000	0.000	1.000	0.000
Ra-a33-66	4	573				0.517		0.078	1.028	0.115	1.168			0.110	0.736	0.050	0.103		1.000	0.000	0.000	1.000	0.000
Ra-a29-66	4	597				0.489		0.087	1.097	0.132	1.066			0.111	0.738	0.048	0.103		1.000	0.000	0.000	1.000	0.000
Ra-a76-66	4	591				0.402		0.080	1.063	0.122	1.064			0.114	0.746	0.040	0.101		1.000	0.000	0.000	1.000	0.000
5-4x	4	601				0.465		0.091	0.971	0.158	1.146			0.107	0.645	0.072	0.176		1.000	0.000	0.000	1.000	0.000
5-4y	4	588				0.454		0.081	1.023	0.137	1.146			0.097	0.654	0.078	0.171		1.000	0.000	0.000	1.000	0.000
44988	4	574				0.518		0.080	0.952	0.130	1.197			0.109	0.680	0.039	0.172		1.000	0.000	0.000	1.000	0.000
45202	4	583				0.483		0.064	0.973	0.130	1.215			0.114	0.690	0.062	0.135		1.000	0.000	0.000	1.000	0.000
61-2	4	575				0.482		0.071	0.976	0.121	1.216			0.103	0.647	0.042	0.208		1.000	0.000	0.000	1.000	0.000
1	4	581				0.483		0.081	0.940	0.125	1.208			0.107	0.663	0.077	0.154		1.000	0.000	0.000	1.000	0.000
N43	4	571				0.477		0.068	0.922	0.138	1.248			0.093	0.619	0.084	0.203		1.000	0.000	0.000	1.000	0.000
N147	4	577				0.490		0.087	1.062	0.144	1.032			0.091	0.711	0.061	0.137		1.000	0.000	0.000	1.000	0.000
42-5	4	576				0.458		0.097	1.104	0.165	1.028			0.085	0.693	0.047	0.175		1.000	0.000	0.000	1.000	0.000
N91C	4	577				0.468		0.089	1.126	0.126	1.014			0.092	0.738	0.051	0.119		1.000	0.000	0.000	1.000	0.000
Ra-a69-66	4	585				0.519		0.086	1.185	0.117	0.970			0.099	0.784	0.037	0.080		1.000	0.000	0.000	1.000	0.000
44971	4	579				0.470		0.103	1.041	0.260	0.942			0.086	0.724	0.016	0.174		1.000	0.000	0.000	1.000	0.000
45130	4	613				0.472		0.121	1.119	0.182	0.946			0.107	0.742	0.043	0.109		1.000	0.000	0.000	1.000	0.000
53	4	591				0.495		0.096	1.118	0.117	1.048			0.112	0.757	0.034	0.097		1.000	0.000	0.000	1.000	0.000
137-1	4	584				0.482		0.101	1.089	0.135	1.035			0.110	0.776	0.037	0.078		1.000	0.000	0.000	1.000	0.000
44991	4	617				0.516		0.139	1.050	0.079	1.052			0.117	0.667	0.071	0.145		1.000	0.000	0.000	1.000	0.000
N41	4	596				0.485		0.100	0.982	0.103	1.209			0.130	0.680	0.032	0.159		1.000	0.000	0.000	1.000	0.000
N102	4	579				0.464		0.092	1.185	0.124	0.960			0.098	0.798	0.024	0.080		1.000	0.000	0.000	1.000	0.000
45050	4	613				0.524		0.106	0.990	0.135	1.082			0.114	0.659	0.073	0.154		1.000	0.000	0.000	1.000	0.000
D237x	4	600				0.471		0.101	1.174	0.124	0.994			0.110	0.771	0.032	0.088		1.000	0.000	0.000	1.000	0.000
D237y	4	591				0.474		0.111	1.113	0.118	1.017			0.112	0.768	0.032	0.089		1.000	0.000	0.000	1.000	0.000
D267x	4	618				0.477		0.090	1.163	0.174	0.927			0.101	0.735	0.052	0.112		1.000	0.000	0.000	1.000	0.000
D267y	4	611				0.449		0.104	1.198	0.179	0.934			0.105	0.771	0.032	0.093		1.000	0.000	0.000	1.000	0.000
D330x	4	607				0.464		0.094	1.069	0.219	0.982			0.110	0.747	0.034	0.109		1.000	0.000	0.000	1.000	0.000
D330y	4	609				0.467		0.089	1.066	0.218	1.012			0.113	0.747	0.038	0.102		1.000	0.000	0.000	1.000	0.000
Ra-a93-66	4	606				0.470		0.094	1.059	0.187	1.069			0.108	0.708	0.069	0.116		1.000	0.000	0.000	1.000	0.000
Ra-b42-66	4	594				0.524		0.095	1.114	0.124	1.084			0.112	0.735	0.040	0.114		1.000	0.000	0.000	1.000	0.000
Ra-c35-66	4	586				0.497		0.101	1.133	0.126	1.017			0.104	0.762	0.033	0.101		1.000	0.000	0.000	1.000	0.000
Ra-a95-66	4	586				0.535		0.092	1.185	0.076	0.980			0.096	0.759	0.055	0.089		1.000	0.000	0.000	1.000	0.000
Ra-b4-66	4	597				0.528		0.107	1.185	0.076	1.014			0.099	0.720	0.064	0.117		1.000	0.000	0.000	1.000	0.000
Ra-b41-66	4	605				0.500		0.101	1.179	0.062	1.032			0.106	0.709	0.058	0.127		1.000	0.000	0.000	1.000	0.000
O-J-73	4	614				0.493		0.098	1.208	0.165	0.883			0.098	0.755	0.039	0.108		1.000	0.000	0.000	1.000	0.000
O-K-15	4	591				0.523		0.086	1.204	0.119	0.958			0.083	0.675	0.033	0.209		1.000	0.000	0.000	1.000	0.000
Ra-b56-66	4	610				0.497		0.105	1.194	0.133	0.937			0.103	0.749	0.041	0.107		1.000	0.000	0.000	1.000	0.000
Ra-b86-66	4	603				0.490		0.106	1.183	0.075	1.054			0.104	0.701	0.056	0.139		1.000	0.000	0.000	1.000	0.000
Ra-c62-66	4	611				0.544		0.097	1.071	0.166	0.988			0.112	0.717	0.032	0.139		1.000	0.000	0.000	1.000	0.000
O-J-65	4	604				0.458		0.089	1.217	0.136	0.916			0.101	0.787	0.041	0.071		1.000	0.000	0.000	1.000	0.000
O-L-10	4	606				0.458		0.118	1.242	0.154	0.874			0.089	0.745	0.044	0.122		1.000	0.000	0.000	1.000	0.000
Ra-b53-66	4	589				0.448		0.089	1.019	0.139	1.129			0.105	0.693	0.068	0.134		1.000	0.000	0.000	1.000	0.000
50-3	4	615				0.486		0.149	1.125	0.199	0.899			0.102	0.757	0.024	0.118		1.000	0.000	0.000	1.000	0.000
63-1	4	611				0.464		0.130	1.099	0.225	0.918			0.093	0.702	0.050	0.155		1.000	0.000	0.000	1.000	0.000
D241	4	602				0.484		0.131	1.169	0.144	0.936			0.094	0.696	0.017	0.194		1.000	0.000	0.000	1.000	0.000
D202x	4	616				0.463		0.134	1.151	0.143	0.973			0.101	0.678	0.033	0.188		1.000	0.000	0.000	1.000	0.000
D202y	4	619				0.487		0.118	1.145	0.141	0.972			0.100	0.672	0.053	0.175		1.000	0.000	0.000	1.000	0.000
112-2	4	605				0.632		0.108	1.077	0.120	0.914			0.098	0.682	0.034	0.187		1.000	0.000	0.000	1.000	0.000
45126	4	591				0.504		0.125	1.113	0.166	0.989			0.096	0.707	0.023	0.174		1.000	0.000	0.000	1.000	0.000
D176x	4	602				0.475		0.103	1.158	0.143	1.018			0.100	0.702	0.033	0.165		1.000	0.000	0.000	1.000	0.000
D176y	4	615				0.441		0.141	1.140	0.142	1.014			0.100	0.671	0.059	0.169		1.000	0.000	0.000	1.000	0.000
140-1	4	629				0.444		0.135	1.051	0.216	1.018			0.114	0.666	0.030	0.189		1.000	0.000	0.000	1.000	0.000
D186	4	605				0.454		0.128	1.108	0.137	1.062			0.106	0.683	0.042	0.169		1.000	0.000	0.000	1.000	0.000
44956	4	626				0.497		0.123	1.005	0.177	1.031			0.106	0.614	0.053	0.227		1.000	0.000	0.000	1.000	0.000
D129A	4	600				0.473		0.124	1.092	0.134	1.052			0.100	0.650	0.019	0.231		1.000	0.000	0.000	1.000	0.000
44934	4	639				0.450		0.160	0.999	0.137	1.090			0.132	0.636	0.057	0.175		1.000	0.000	0.000	1.000	0.000
47-1	4	614				0.517		0.085	1.013	0.151	1.160			0.120	0.655	0.055	0.170		1.000	0.000	0.000	1.000	0.000
4	4	608				0.539		0.133	0.966	0.120	1.132			0.116	0.645	0.078	0.161		1.000	0.000	0.000	1.000	0.000
D171	4	624				0.481		0.106	0.950	0.117	1.229			0.117	0.562	0.071	0.250		1.000	0.000	0.000	1.000	0.000
O-C-30	4	603				0.446		0.146	1.160	0.101	1.047			0.105	0.688	0.030	0.178		1.000	0.000	0.000	1.000	0.000
O-C-14	4	619				0.492		0.136	1.172	0.101	0.982			0.116	0.715	0.022	0.147		1.000	0.000	0.000	1.000	0.000
O-C-17	4	614				0.440		0.125	1.197	0.195	0.843			0.095	0.759	0.029	0.117		1.000	0.000	0.000	1.000	0.000
O-C-18	4	618				0.469		0.124	1.126	0.247	0.879			0.099	0.737	0.031	0.133		1.000	0.000	0.000	1.000	0.000
O-C-26	4	614				0.426		0.144	1.123	0.098	1.043			0.112	0.672	0.030	0.186		1.000	0.000	0.000	1.000	0.000
O-K-10	4	603				0.552		0.149	1.245	0.123	0.824			0.084	0.729	0.036	0.151		1.000	0.000	0.000	1.000	0.000
O-K-29	4	630				0.472		0.138	1.249	0.154	0.829			0.101	0.754	0.028	0.117		1.000	0.000	0.000	1.000	0.000
77-2	4	626				0.492		0.145	1.035	0.178	1.010			0.124	0.703	0.026	0.147		1.000	0.000	0.000	1.000	0.000
77-3	4	632				0.475		0.139	1.064	0.183	1.031			0.126	0.697	0.026	0.151		1.000	0.000	0.000	1.000	0.000
90	4	631				0.452		0.168	1.065	0.183	0.980			0.115	0.672	0.021	0.192		1.000	0.000	0.000	1.000	0.000
76	4	633				0.444		0.178	1.068	0.184	0.968			0.115	0.678	0.027	0.181		1.000	0.000	0.000	1.000	0.000
56	4	636				0.437		0.168	1.094	0.188	0.943			0.114	0.702	0.040	0.143		1.000	0.000	0.000	1.000	0.000
91	4	637				0.434		0.197	1.112	0.191	0.859			0.104	0.689	0.025	0.181		1.000	0.000	0.000	1.000	0.000
73	4	627				0.475		0.210	1.069	0.141	0.940			0.111	0.668	0.033	0.188		1.000	0.000	0.000	1.000	0.000
87	4	645				0.492		0.177	1.098	0.146	0.940			0.129	0.725	0.062	0.084		1.000	0.000	0.000	1.000	0.000
143	4	642				0.467		0.171	1.112	0.147	0.939			0.133	0.744	0.029	0.094		1.000	0.000	0.000	1.000	0.000
145	4	633				0.507		0.203	1.128	0.150	0.832			0.117	0.759	0.023	0.102		1.000	0.000	0.000	1.000	0.000
86	4	649				0.475		0.182	1.127	0.150	0.940			0.149	0.772	0.016	0.063		1.000	0.000	0.000	1.000	0.000
