# Supplementary material for: A new activity model for biotite and its application
Source: Contrib Mineral Petrol. 2024 Sep 30;179(10):93. doi: 10.1007/s00410-024-02173-6 (PMC11452188; doi:10.1007/s00410-024-02173-6)
Supplement: Supplementary file 3 — Supplementary file3 (PDF 202 KB) [file 410_2024_2173_MOESM3_ESM.pdf]

Edgar Dachs and Artur Benisek (2024): "A new activity model for biotite and its application"
(Contributions to Mineralogy and Petrology, in press)
Department of Chemistry and Physics of Materials, University of Salzburg
Jakob-Haringerstrasse 2a, A-5020 Salzburg, Austria
E-mail: edgar.dachs@plus.ac.at

Supplementary Table 3 Perple_X-computed compositions of biotite and garnet in experimental Fe-Mg exchange experiments between garnet and biotite of Ferry and Spear (1978 - FS78) and Gessmann et al. (1997 - G97), using the biotite activity models Bio(D) and its ideal Fe-Mg mixing version Bio(Did) of this study and models Bi(W) and Bio(TCC), published by White et al. (2000, 2014) and Tajcmanova et al. (2009) for biotite and Gt(W) for garnet (White et al., 2014). Bulk-compositions used in Perple_X calculations are given in supplementary Table 5 of this study.
Pressure in both experimental studies was P = 2.07 kbar; Oxygen fugacity in FS78-experiments was controlled by the C-CH4 buffer, in G97-experiments by the QFM- and the CCO buffers.
Garnet composition, due to the high grt/bio ratio in the experimental setup, remained nearly unchanged and was X_{Fe} = 0.9 in FS78 and 0.8 in G97 -runs.
Experimentally determined biotite compositions (X_{Fe} for FS78, X_{Fe} and Al^{VI} for G97 experiments) are given for comparison with computed values. $X_{Fe}^{(2+)}$ with best agreement to the experimental value is marked in bold.
Al^{IV} : tetrahedral Al; Al^{VI}: octahedral Al; $Al^{VI,ex}$: octahedral excess-Al;
$X_{Fe}^{2+} = Fe^{2+}/(Fe^{2+} + Mg)$; $X_{Fe} = (Fe^{2+} + Fe^{3+})/(Fe^{2+} + Fe^{3+} + Mg)$

Ferry and Spear (1978):			biotite												garnet	
		activity	computed composition (atoms per formula unit - 11 oxygens)											experimental	computed comp.	
label/bulk	T (°C)	model	Si	Al^{IV}	Al^{VI}	$Al^{VI,ex}$	Fe^{2+}	Fe^{3+}	Mg	K	X_{Fe}^{2+}	X_{Fe}	X_{Fe}^{3+}	X_{Fe}	X_{Alm}	X_{Kho1}
118	550	Bio(D)	2.564	1.436	0.425	0.043	1.446	0.076	1.032	0.978	**0.584**	**0.596**	0.050	**0.587**	0.897	0.007
Alm90Ann50		Bio(Did)	2.553	1.447	0.430	0.045	1.482	0.085	0.981	0.978	**0.602**	**0.615**	0.054		0.896	0.008
		Bi(W)	2.670	1.330	0.247		1.808	0.083	0.861	1.000	0.677	0.687	0.044		0.895	0.007
		Bio(TCC)	2.572	1.428	0.357		1.693	0.071	0.879	1.000	0.658	0.667	0.040		0.895	0.008
126	550	Bio(D)	2.560	1.440	0.387	0.044	1.469	0.075	1.004	0.978	**0.594**	**0.606**	0.049	**0.620**	0.902	0.007
Alm90Ann75		Bio(Did)	2.551	1.449	0.432	0.113	1.510	0.085	0.951	0.978	**0.614**	**0.627**	0.053		0.901	0.008
		Bi(W)	2.668	1.332	0.248		1.847	0.084	0.821	1.000	0.692	0.702	0.043		0.900	0.007
		Bio(TCC)	2.565	1.435	0.364		1.729	0.071	0.836	1.000	0.674	0.683	0.040		0.900	0.008
137	599	Bio(D)	2.583	1.417	0.428	0.050	1.537	0.064	0.946	0.975	**0.619**	**0.629**	0.040	**0.608**	0.896	0.008
Alm90Ann50		Bio(Did)	2.577	1.423	0.426	0.051	1.575	0.073	0.900	0.974	**0.636**	**0.647**	0.045		0.896	0.010
		Bi(W)	2.639	1.361	0.273		1.869	0.088	0.770	1.000	0.708	0.718	0.045		0.895	0.008
		Bio(TCC)	2.534	1.466	0.395		1.767	0.071	0.767	1.000	0.697	0.705	0.039		0.895	0.010
139	599	Bio(D)	2.582	1.418	0.430	0.050	1.563	0.064	0.917	0.975	**0.630**	**0.640**	0.040	**0.645**	0.901	0.008
Alm90Ann75		Bio(Did)	2.576	1.424	0.428	0.051	1.602	0.073	0.870	0.958	**0.648**	**0.658**	0.044		0.901	0.010
		Bi(W)	2.638	1.362	0.273		1.903	0.089	0.734	1.000	0.722	0.731	0.045		0.900	0.008
		Bio(TCC)	2.528	1.472	0.401		1.799	0.071	0.729	1.000	0.712	0.720	0.038		0.900	0.010
135	651	Bio(D)	2.606	1.394	0.423	0.056	1.626	0.056	0.868	0.972	**0.652**	**0.660**	0.033	**0.661**	0.895	0.010
Alm90Ann50		Bio(Did)	2.599	1.401	0.423	0.058	1.661	0.064	0.824	0.971	**0.668**	**0.677**	0.037		0.895	0.012
		Bi(W)	2.609	1.391	0.298		1.916	0.093	0.693	1.000	0.734	0.744	0.046		0.894	0.011
		Bio(TCC)	2.502	1.498	0.427		1.826	0.071	0.676	1.000	0.730	0.737	0.037		0.894	0.012
138	651	Bio(D)	2.605	1.395	0.424	0.057	1.652	0.056	0.840	0.972	**0.663**	**0.670**	0.033	**0.679**	0.900	0.010
Alm90Ann75		Bio(Did)	2.598	1.402	0.396	0.058	1.687	0.064	0.795	0.971	**0.680**	**0.688**	0.037		0.900	0.012
		Bi(W)	2.608	1.392	0.298		1.948	0.094	0.660	1.000	0.747	0.756	0.046		0.899	0.010
		Bio(TCC)	2.497	1.503	0.432		1.855	0.071	0.642	1.000	0.743	0.750	0.037		0.899	0.012
125	698	Bio(D)	2.625	1.375	0.418	0.062	1.695	0.049	0.807	0.969	**0.678**	**0.684**	0.028	**0.690**	0.895	0.013
Alm90Ann50		Bio(Did)	2.618	1.382	0.420	0.063	1.732	0.057	0.764	0.969	**0.694**	**0.701**	0.032		0.894	0.014
		Bi(W)	2.586	1.414	0.317		1.950	0.097	0.636	1.000	0.754	0.763	0.047		0.894	0.011
		Bio(TCC)	2.479	1.521	0.449		1.867	0.072	0.612	1.000	0.753	0.760	0.037		0.893	0.014
128	698	Bio(D)	2.645	1.355	0.363	0.059	1.743	0.051	0.790	0.963	**0.688**	**0.694**	0.029	**0.704**	0.899	0.013
Alm90Ann75		Bio(Did)	2.656	1.344	0.369	0.057	1.780	0.061	0.761	0.972	**0.701**	**0.708**	0.033		0.898	0.017
		Bi(W)	2.612	1.388	0.290		1.998	0.098	0.614	1.000	0.765	0.774	0.047		0.898	0.013
		Bio(TCC)	2.533	1.467	0.388		1.916	0.079	0.617	1.000	0.756	0.764	0.040		0.895	0.017
123	749	Bio(D)	2.648	1.352	0.388	0.057	1.762	0.050	0.772	0.972	**0.695**	**0.701**	0.027	**0.695**	0.891	0.015
Alm90Ann50		Bio(Did)	2.626	1.374	0.415	0.064	1.770	0.054	0.729	0.969	**0.708**	**0.714**	0.030		0.891	0.018
		Bi(W)	2.588	1.412	0.310		1.999	0.101	0.589	1.000	0.772	0.781	0.048		0.893	0.015
		Bio(TCC)	2.491	1.509	0.432		1.918	0.077	0.573	1.000	0.770	0.777	0.039		0.893	0.018
89	738	Bio(D)	2.685	1.315	0.326	0.059	1.859	0.047	0.709	0.971	**0.724**	**0.729**	0.025	**0.730**	0.900	0.017
Alm90Ann100		Bio(Did)	2.717	1.283	0.305	0.054	1.857	0.059	0.752	0.973	**0.712**	**0.718**	0.031		0.895	0.024
		Bi(W)	2.635	1.365	0.262		2.078	0.103	0.557	1.000	0.789	0.797	0.047		0.902	0.017
		Bio(TCC)	2.586	1.414	0.328		1.972	0.087	0.614	1.000	0.763	0.770	0.042		0.895	0.024
116	799 / 2.9 kbar	Bio(D)	2.686	1.314	0.354	0.056	1.832	0.045	0.741	0.972	**0.712**	**0.717**	0.024	**0.710**	0.890	0.020
Alm90Ann50	799 / 2.4 kbar	Bio(Did)	Bio not stable													
	799 / 2.7 kbar	Bi(W)	2.599	1.401	0.296		2.033	0.105	0.566	1.000	0.782	0.791	0.049		0.891	0.042
	799 / 2.7 kbar	Bio(TCC)	2.534	1.466	0.377		1.966	0.089	0.568	1.000	0.776	0.783	0.043		0.889	0.027
112	799 / 2.8 kbar	Bio(D)	Bio not stable											**0.750**		
Alm90Ann100	799 / 2.7 kbar	Bio(Did)	Bio not stable													
	799 / 3.0 kbar	Bi(W)	2.663	1.337	0.231		2.129	0.107	0.534	1.000	0.800	0.807	0.048		0.899	0.023
	799 / 2.7 kbar	Bio(TCC)	Bio not stable													

Gessmann et al. (1997)			biotite													garnet		
		activity	computed composition (atoms per formula unit - 11 oxygens)											experimental		computed composition		experimental
run/bulk	T (°C)	model	Si	Al^{IV}	Al^{VI}	$Al^{VI,ex}$	Fe^{2+}	Fe^{3+}	Mg	K	X_{Fe}^{2+}	X_{Fe}	X_{Fe}^{3+}	X_{Fe}	Al^{VI}	X_{Alm}	X_{Kho1}	X_{Fe}
80/1 - QFM	650	Bio(D)	2.644	1.356	0.364	0.050	1.174	0.068	1.369	0.975	**0.462**	**0.476**	0.055	**0.493**	0.276	0.778	0.013	0.779
Alm80Ann10		Bio(Did)	2.657	1.343	0.352	0.050	1.202	0.067	1.354	0.975	**0.470**	**0.484**	0.053			0.777	0.013	
		Bi(W)	2.651	1.349	0.257		1.274	0.093	1.377	1.000	0.480	0.498	0.068			0.776	0.012	
		Bio(TCC)	2.622	1.378	0.312		1.232	0.066	1.390	1.000	0.470	0.483	0.051			0.777	0.013	
80/12 - CCO	650	Bio(D)	2.655	1.345	0.360	0.049	1.234	0.058	1.324	0.976	**0.482**	**0.494**	0.045	**0.435**	0.249	0.793	0.011	0.783
Alm80Ann40		Bio(Did)	2.667	1.333	0.350	0.049	1.262	0.057	1.307	0.975	**0.491**	**0.502**	0.043			0.793	0.011	
		Bi(W)	2.669	1.331	0.251		1.357	0.080	1.312	1.000	0.508	0.523	0.056			0.791	0.011	
		Bio(TCC)	2.632	1.368	0.311		1.303	0.057	1.329	1.000	0.495	0.506	0.042			0.792	0.011	
80/9 - CCO	700	Bio(D)	2.686	1.314	0.314	0.053	1.359		1.222	0.974	**0.527**	**0.527**	0.000	**0.464**	0.339	0.803	0.014	0.802
Alm80Ann65		Bio(Did)	2.695	1.305	0.307	0.053	1.383	0.051	1.207	0.974	**0.534**	**0.543**	0.035			0.803	0.014	
		Bi(W)	2.649	1.351	0.264		1.486	0.086	1.163	1.000	0.561	0.575	0.055			0.801	0.013	
		Bio(TCC)	2.609	1.391	0.330		1.433	0.061	1.176	1.000	0.549	0.560	0.041			0.802	0.014	
80/6 - QFM	700	Bio(D)	2.664	1.336	0.358	0.055	1.296	0.061	1.258	0.972	**0.507**	**0.519**	0.045	**0.462**	0.471	0.790	0.015	0.788
Alm80Ann40		Bio(Did)	2.671	1.329	0.354	0.056	1.321	0.059	1.239	0.972	**0.516**	**0.527**	0.043			0.790	0.015	
		Bi(W)	2.615	1.385	0.285		1.403	0.100	1.212	1.000	0.536	0.554	0.067			0.789	0.015	
		Bio(TCC)	2.593	1.407	0.338		1.365	0.070	1.228	1.000	0.527	0.539	0.049			0.789	0.015	
80/3 - QFM	750	Bio(D)	2.702	1.298	0.332	0.058	1.418	0.054	1.167	0.971	**0.548**	**0.558**	0.037	**0.499**	0.357	0.801	0.019	0.809
Alm80Ann65		Bio(Did)	2.710	1.290	0.326	0.059	1.440	0.053	1.152	0.971	**0.556**	**0.564**	0.035			0.800	0.019	
		Bi(W)	2.634	1.366	0.257		1.602	0.109	1.032	1.000	0.608	0.624	0.064			0.809	0.019	
		Bio(TCC)	2.580	1.420	0.347		1.484	0.073	1.096	1.000	0.575	0.587	0.047			0.800	0.019	
80/4 - QFM	750	Bio(D)	2.701	1.299	0.334	0.059	1.362	0.053	1.221	0.971	**0.527**	**0.537**	0.038	**0.473**	0.354	0.782	0.018	0.781
Alm80Ann25		Bio(Did)	2.711	1.289	0.325	0.059	1.385	0.052	1.209	0.971	**0.534**	**0.543**	0.036			0.782	0.018	
		Bi(W)	2.626	1.374	0.271		1.469	0.104	1.156	1.000	0.560	0.576	0.066			0.781	0.018	
		Bio(TCC)	2.583	1.417	0.346		1.420	0.071	1.163	1.000	0.550	0.562	0.048			0.782	0.018	
80/11 - CCO	800 / 2.8 kbar	Bio(D)	2.718	1.262	0.313	0.060	1.487	0.040	1.131	0.970	**0.568**	**0.575**	0.026	**0.470**	0.408	0.807	0.018	0.792
Alm80Ann65		Bio(Did)	2.740	1.260	0.317	0.063	1.503	0.038	1.111	0.969	**0.575**	**0.581**	0.025			0.798	0.018	
		Bi(W)	2.633	1.367	0.274		1.646	0.093	0.987	1.000	0.625	0.638	0.053			0.800	0.018	
		Bio(TCC)	2.576	1.424	0.361		1.587	0.063	0.989	1.000	0.616	0.625	0.038			0.800	0.018	
80/10 - CCO	800 / 2.9 kbar	Bio(D)	2.732	1.268	0.324	0.062	1.439	0.038	1.169	0.967	**0.552**	**0.558**	0.026	**0.491**	0.336	0.786	0.017	0.770
Alm80Ann40		Bio(Did)	2.738	1.262	0.321	0.064	1.456	0.037	1.154	0.968	**0.558**	**0.564**	0.025			0.786	0.017	
		Bi(W)	2.633	1.367	0.274		1.645	0.093	0.988	1.000	0.625	0.637	0.054			0.801	0.018	
		Bio(TCC)	2.576	1.424	0.361		1.585	0.063	0.990	1.000	0.616	0.625	0.038			0.802	0.018	
