# Supplementary material for: A new activity model for biotite and its application
Source: Contrib Mineral Petrol. 2024 Sep 30;179(10):93. doi: 10.1007/s00410-024-02173-6 (PMC11452188; doi:10.1007/s00410-024-02173-6)
Supplement: Supplementary file 4 — Supplementary file4 (PDF 230 KB) [file 410_2024_2173_MOESM4_ESM.pdf]

Edgar Dachs and Artur Benisek (2024): "A new activity model for biotite and its application"
(Contributions to Mineralogy and Petrology, in press)
Department of Chemistry and Physics of Materials, University of Salzburg
Jakob-Haringerstrasse 2a, A-5020 Salzburg, Austria
E-mail: edgar.dachs@plus.ac.at

Supplementary Table 4 Macroscopic W_{ij}'s and their decomposition into microscopic interaction parameters *w*'s for the KFMASHTO biotite activity model of this study, with end-members given in Table 1. For macroscopic W_{phlann} and W_{phleas} (bold), corresponding micro-w's have been quantified using *Castep* (Table 5). V = vacancy, Fe3 = Fe^{3+}.

	**n**	1	2	3	4	5	6	7	8	9	10	11	12	13	14	15	16	17	18	19	20	21
	**macro-*W***	***W***	***W***	*W*	*W*	*W*	*W*	*W*	*W*	*W*	*W*	*W*	*W*	*W*	*W*	*W*	*W*	*W*	*W*	*W*	*W*	*W*
	**i**	**phl**	**phl**	phl	phl	ann	ann	ann	eas	eas	obi	phl	ann	eas	obi	pyp	phl	ann	eas	obi	pyp	tbio
	**j**	**ann**	**eas**	obi	pyp	eas	obi	pyp	obi	pyp	pyp	tbio	tbio	tbio	tbio	tbio	fbio	fbio	fbio	fbio	fbio	fbio
	**kJ/mol**	**-2.4**	**18.8**	-0.1	117	-5	-0.4	108	-5	120	120	0	-30									
	multipli-cator	1	1/4	1	1/4	1/4	1	1/4	1/4	1	1/4	1/4	1/4	1/4	1/4	1/4	1/4	1/4	1	1/4	1	1/4
**Site**	**micro-*w***	**same site mixing**																				
A	*w*KV				4			4		1	4					4						
M1	*w*MgFe	1		1									4		4							
	*w*MgAl		4											4								
	*w*MgV				4											4						
	*w*MgFe3																4					4
	*w*FeAl					4			4													
	*w*FeV							4			4											
	*w*FeFe3																	4		4		
	*w*AlV									1												
	*w*AlFe3																		1			
	*w*VFe3																				1	
M2	*w*MgFe	1				4	1						2					4				
	*w*MgAl				4					1	4					2					1	

	*w*MgTi											1	-1	1	1	-1						
	*w*FeAl							4														
	*w*FeTi												2									
	*w*AlTi															2						
T1	*w*AlSi		1		1	1		1	1	1	1			1		1	1	1		1	1	1
OH	*w*OHO											4	4	4	4	4						
**Cross-sites**	**micro-*w***	**cross-site mixing**																				
AM1	*w*KMgVV				4											4						
	*w*KFeVV							4			4											
	*w*KAlVV									1												
	*w*KVVFe3																				-1	
AM2	*w*KMgVAl				4					1	4					4					1	
	*w*KFeVAl							4														
	*w*KMgVTi															-2						
AT1	*w*KAlVSi				2			2		1	2					2					1	
AOH	*w*KOHVO															-4						
M1M2	*w*MgMgFeFe	1											4									
	*w*MgMgVAl				4											4						
	*w*FeFeVAl							4														
	*w*AlMgVAl									1												
	*w*FeMgVAl										4											
	*w*FeMgAlFe					-4																
	*w*MgMgFeTi												-2		-2							
	*w*MgMgAlTi													-2								
	*w*MgMgVTi															-2						
	*w*FeMgFe3Fe																	-4				
	*w*VMgFe3Al																				-1	
	*w*MgMgFe3Ti																					-2
M1T1	*w*MgAlAlSi		-2											-2								
	*w*FeAlAlSi					-2			-2													
	*w*MgAlVSi															2						
	*w*FeAlVSi							2			2											
	*w*AlAlVSi									1												
	*w*MgAlFe3Si																-2					-2
	*w*FeAlFe3Si																	-2		-2		
	*w*VAlFe3Si																				-1	

M1OH	*w*MgOHFeO									-4		-4							
	*w*MgOHAlO										-4								
	*w*MgOHVO												-4						
	*w*MgOHFe3O																		-4
M2T1	*w*MgAlAlSi	2					1	2					2					1	
	*w*FeAlAlSi				2														
	*w*MgAlFeSi		2												2				
	*w*MgAlTiSi										1		-1						1
M2OH	*w*MgOHTiO								2				2						
	*w*MgOHFeO									-4									
	*w*MgOHTiO									2	2	2							2
T1OH	*w*AlOHSiO										2		-2						2
