# Supplementary material for: A new activity model for biotite and its application
Source: Contrib Mineral Petrol. 2024 Sep 30;179(10):93. doi: 10.1007/s00410-024-02173-6 (PMC11452188; doi:10.1007/s00410-024-02173-6)
Supplement: Supplementary file 5 — Supplementary file5 (PDF 131 KB) [file 410_2024_2173_MOESM5_ESM.pdf]

Edgar Dachs and Artur Benisek (2024): "A new activity model for biotite and its application"
(Contributions to Mineralogy and Petrology, in press)
Department of Chemistry and Physics of Materials, University of Salzburg
Jakob-Haringerstrasse 2a, A-5020 Salzburg, Austria
E-mail: edgar.dachs@plus.ac.at

Supplementary Table 5 Bulk compositions (wt.%) used in *Perple_X* calculations

Label	SiO_2	TiO_2	Al_2O_3	FeO	Fe_2O_3	MgO	MnO	CaO	Na_2O	K_2O	H_2O	Ref.	Fig.
HQ-36	57.36	1.26	23.24	8.59		2.72	0.17	0.40	0.48	3.63	1.69	PD93[1)]	4, 5, 7
CEVP	69.99	0.70	12.96	4.83		2.36	0.06	1.67	2.95	2.41	1.43	VM94	4
SBG	63.4	2.50	12.30	7.80		4.70	0.10	2.10	2.00	3.60	1.40	PDB95	4
Alm90Ann50	36.98	0.00	20.71	39.44		2.68	0.00	0.00	0.00	0.19	excess	D21/FS78	6
Alm90Ann75	36.94	0.00	20.69	39.62		2.55	0.00	0.00	0.00	0.19	excess	D21/FS78	6
Alm90Ann100	36.91	0.00	20.67	39.81		2.43	0.00	0.00	0.00	0.19	excess	D21/FS78	6
Alm80Ann10	37.92	0.00	20.91	34.69		5.98	0.00	0.00	0.00	0.50	excess	D21/FS78	6
Alm80Ann25	37.87	0.00	20.88	34.98		5.78	0.00	0.00	0.00	0.49	excess	D21/G97	6
Alm80Ann40	37.81	0.00	20.85	35.26		5.58	0.00	0.00	0.00	0.49	excess	D21/G97	6
Alm80Ann65	37.72	0.00	20.80	35.74		5.25	0.00	0.00	0.00	0.49	excess	D21/G97	6
average pelite	60.77	0.97	18.43	5.11	1.91	2.58	0.12	1.29	1.79	3.80	excess	FP21	3
16	62.56	0.85	14.15	5.53	1.14	2.54	0.16	3.30	1.59	3.87	2.20	M70	8, S1a[2)]
18	76.17	0.46	10.9	2.16	0.67	0.84	0.05	1.63	4.57	1.15	0.70	M70	8, S1b[2)]
980A	52.43	0.92[3)]	20.78	8.42		3.60	0.16	2.70	2.59	4.68	excess	T01/F80	S2a-c[2)]
X567	49.56	1.33	27.86	13.90		3.10	0.09	0.79	0.90	2.47	ex/red	PdW01	9
XRFA1	57.43	1.09	23.92	5.8		1.46	0.09	0.21	0.19	4.82	5.00	L20	

[1)] PD93: Patino Douce et al. (1993): peraluminous metapelite; D21: Dachs et al. (2021): bulks for Ferry and Spear (1978 – FS78) and Gessmann et al. (1997 – G97) Fe-Mg exchange experiments between garnet and biotite; FP21: Forshaw and Pattison (2021): metapelite database average; M70: Mather (1970): 16: metapelite with 1.57 wt.% CO_2; 18: metagreywacke with assumed 0.3 wt.% CO_2; T01: Tinkham et al (2001): metapelite with mineral compositions reported in Ferry (1980 – F80); PdW01: Pitra and de Waal (2001): metapelite; L20: Li et al. (2020): pelitic micaschist.
[2)] Fig. S1a, Fig. S1b, or Figs. S2a-c in Online Resource 7.
[3)] mean value of samples from the staurolite-andalusite zone (Ferry 1982).
