# Supplementary material for: A new activity model for biotite and its application
Source: Contrib Mineral Petrol. 2024 Sep 30;179(10):93. doi: 10.1007/s00410-024-02173-6 (PMC11452188; doi:10.1007/s00410-024-02173-6)
Supplement: Supplementary file 6 — Supplementary file6 (PDF 193 KB) [file 410_2024_2173_MOESM6_ESM.pdf]

Edgar Dachs and Artur Benisek (2024): "A new activity model for biotite and its application"
(Contributions to Mineralogy and Petrology, in press)
Department of Chemistry and Physics of Materials, University of Salzburg
Jakob-Haringerstrasse 2a, A-5020 Salzburg, Austria
E-mail: edgar.dachs@plus.ac.at

Supplementary Table 6 Predicted versus measured compositions of selected minerals from test samples. Calculations were done with *Perple_X* (program *Werami*) with thermodynamic standard state data of biotite endmembers and the activity model Bio(D) from this study (Tables 2, 3), compared to calculations with *Perple_X*-implemented biotite models Bi(W) (White et al. 2014) and Bio(TCC) (Tajčmanova et al. 2009). Other solid-solution models used are given in the text, bulk-rock compositions are given in supplementary Table 5.

sample / ref.	P	T	biotite	biotite composition										other compositions				
assemblage	kbar	°C	model	Si	Al^{IV}	Al^{VI}	Al^{VI}_{ex}	Ti	Fe^{2+}	Fe^{3+}	Mg	K	$X_{Fe^{2+}}$					
**16 / M70**[1)]	4	350												**chl**[2)]	**chl/bio**	**phe**		**chl/phe**
														$X_{Fe^{2+}}$	K_D	Si	$X_{Fe^{2+}}$	K_D
bio-chl-phe-			**measured**	**2.77**	**1.23**	**0.31**	**0.08**	**0.12**	**1.41**	**0.09**	**0.85**	**0.84**	**0.62**	**0.60**	**0.89**	**3.21**	**0.41**	**2.17**
plag-qtz-cal			Bio(D)	2.68	1.32	0.26	0.00	0.04	1.53	0.07	1.10	1.00	0.58	0.55	0.89	3.12	0.44	1.55
			Bi(W)	2.94	1.06	0.05		0.04	1.82	0.01	1.08	1.00	0.63	0.43	0.44	3.04	0.40	1.11
			Bio(TCC)	2.92	1.08	0.06		0.06	1.71	0.02	1.15	1.00	0.60	0.52	0.74	3.05	0.42	1.49
**18 / M70**[1)]	4	410	**measured**	**2.69**	**1.31**	**0.25**	**-0.06**	**0.11**	**1.43**	**0.07**	**0.97**	**0.87**	**0.60**	**0.56**	**0.88**	**3.19**	**0.35**	**2.35**
bio-chl-phe-			Bio(D)	2.75	1.25	0.21	0.00	0.04	1.65	0.06	1.01	0.99	0.62	0.60	0.94	3.17	0.51	1.47
plag-qtz-cal			Bi(W)	2.95	1.05	0.04		0.04	1.88	0.01	1.01	1.00	0.65	0.47	0.48	3.11	0.42	1.24
			Bio(TCC)	2.91	1.09	0.07		0.06	1.79	0.02	1.04	1.00	0.63	0.56	0.75	3.10	0.48	1.40
**980A /**	4	560												**stau**	**plag**		**grt**	
**T01**[1)]**, F80**[1)]														$X_{Fe^{2+}}$	X_{Ca}	$X_{Fe^{2+}}$	X_{Ca}	X_{Mn}
grt-bio-stau-			**measured**			**0.52**		**0.09**		**nd**			**0.52**	**0.84**	**0.35**	**0.89**	**0.06**	**0.12**
phe-and-qtz			Bio(D)	2.48	1.52	0.56	0.04	0.12	1.24	0.04	0.99	0.97	0.56	0.80	0.37	0.88	0.05	0.29
			Bi(W)	2.65	1.35	0.29		0.08	1.38	0.06	1.18	1.00	0.54	0.77	0.38	0.86	0.05	0.29
			Bio(TCC)	2.50	1.50	0.47		0.07	1.30	0.04	1.04	1.00	0.56	0.76	0.37	0.86	0.05	0.34
**X567 /**	2	665												**crd**	**plag**		**grt**	
**PdW01**[1)]														$X_{Fe^{2+}}$	X_{Ca}	$X_{Fe^{2+}}$	X_{Ca}	X_{Mn}
crd-bio-kfsp-			**measured**	**2.53**	**1.47**	**0.48**	**0.01**	**0.19**	**1.73**	**nd**	**0.48**	**0.89**	**0.78**	**0.65**	**0.36**	**0.92**	**0.03**	**0.01**
plag-qtz±grt			Bio(D)	2.55	1.45	0.48	0.03	0.19	1.73	0.05	0.53	0.97	0.77	0.68	0.37	0.95	0.02	0.04
			Bio(Did)[3)]	2.49	1.51	0.55	0.04	0.25	1.72	0.04	0.42	0.97	0.81	0.63	0.32			
			Bi(W)	2.65	1.35	0.26		0.12	2.07	0.09	0.46	1.00	0.82	0.67	0.38			
		730	Bio(TCC)	2.46	1.54	0.48		0.25	1.81	0.06	0.40	1.00	0.82	0.67	0.37			

**16Slo12 / L20[1)]**	9	630												**stau**		**phe**
														**$X_{Fe^{2+}}$**	**Si**	**$X_{Fe^{2+}}$**
bio+stau+ru			**measured**	**2.71**	**1.29**	**0.54**	**0.24**	**0.08**	**0.91**	**nd**	**1.34**	**0.82**	**0.40**	**0.62**	**3.08**	**0.44**
incl. in grt			Bio(D)	2.63	1.37	0.47	0.10	0.11	1.17	0.00	1.21	0.97	0.49	0.72	3.08	0.40
phe_1 in			Bi(W)	2.78	1.22	0.22		0.10	1.29	0.00	1.38	1.00	0.48	0.72	3.08	0.41
matrix			Bio(TCC)	2.61	1.39	0.39		0.17	1.35	0.00	1.09	1.00	0.55	0.77	3.06	0.46
	7	630	Bio(D)	2.58	1.42	0.52	0.10	0.14	1.30	0.00	1.00	0.97	0.56	0.79	3.03	0.48
			Bi(W)	2.73	1.27	0.27		0.11	1.48	0.00	1.15	1.00	0.56	0.78	3.04	0.47
			Bio(TCC)	2.52	1.48	0.48		0.18	1.44	0.00	0.89	1.00	0.62	0.80	3.01	0.50

[1)] M70: Mather (1970); F80: Ferry (1980); T01: Tinkham et al. (2001); PdW01: Pitra and de Waal (2001); L20: Li et al. (2020).

[2)] mineral abbreviations: chl: chlorite; bio: biotite; phe: phengite; stau: staurolite; plag: plagioclase; kfsp: kalifeldspar; grt: garnet; ru: rutile; crd: cordierite; qtz: quartz; $K_D = (Fe/Mg)^{chl}/(Fe/Mg)^{bio}$; nd: not determined.

[3)] simplified version of Bio(D) assuming ideal Fe-Mg mixing.
