# Supplementary material for: A new activity model for biotite and its application
Source: Contrib Mineral Petrol. 2024 Sep 30;179(10):93. doi: 10.1007/s00410-024-02173-6 (PMC11452188; doi:10.1007/s00410-024-02173-6)
Supplement: Supplementary file 7 — Supplementary file7 (PDF 3240 KB) [file 410_2024_2173_MOESM7_ESM.pdf]

**Edgar Dachs and Artur Benisek (2024): "A new activity model for biotite and its application"**
(Contributions to Mineralogy and Petrology, in press)
Department of Chemistry and Physics of Materials, University of Salzburg
Jakob-Haringerstrasse 2a, A-5020 Salzburg, Austria
E-mail: edgar.dachs@plus.ac.at

**Online Resource 7** *Perple_X* raw pseudosections of test samples.

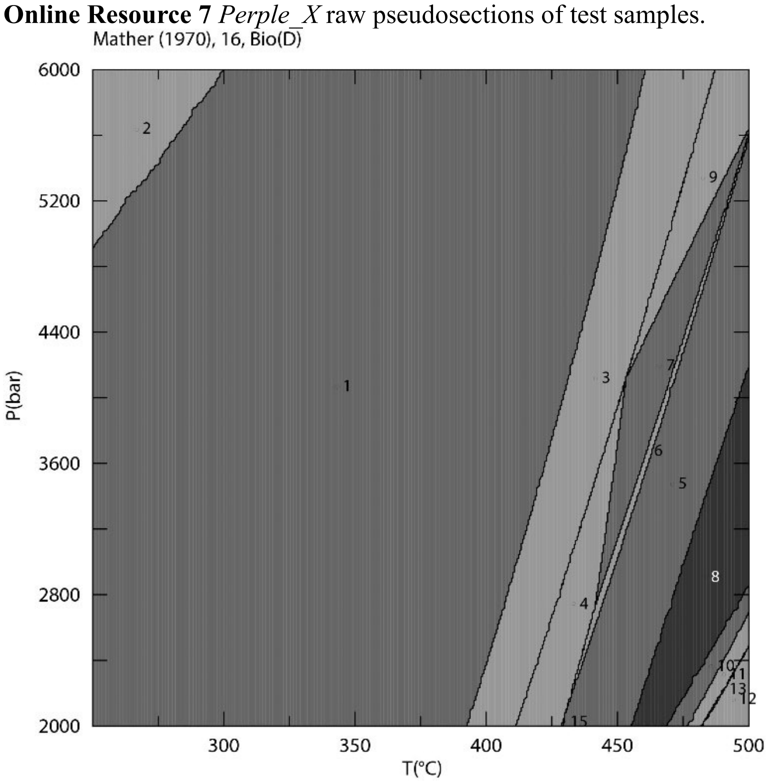


1 - Bio Chl Phe Ep Carb sph ab q CH4
2 - Bio Chl Phe Ep Carb Carb sph ab q CH4
3 - Bio Chl Phe Ep Carb sph ab q H2O CH4
4 - Bio Chl Phe Pl Carb sph ab q H2O CH4
5 - Bio Phe Pl Carb sph q ru H2O CH4
6 - Bio Chl Phe Pl Carb sph q ru H2O CH4
7 - Bio Chl Phe Pl Carb sph q H2O CH4
8 - Bio Phe Pl Carb q ru H2O CH4
9 - Bio Chl Phe Pl Ep Carb sph q H2O CH4
10 - Bio Phe Pl Kfsp Carb q ru H2O CH4
11 - Bio Phe Pl Kfsp Carb q ru H2O CO2 CH4
12 - Bio feldspar Pl Kfsp Carb q ru H2O CO2 CH4

**Fig. S1a** *Perple_X* raw-pseudosection for sample 16 (Mather, 1970), computed with Bio(D). Bulk composition is given in supplementary Table 5. $H_2O$ and $CO_2$ contents were 2.2 and 1.57 wt.%, $O_2$ was assumed to be 0.015 wt.%.
$K_D = (Fe/Mg)_{Chl}/(Fe/Mg)_{Bio} = 0.89$ in this sample and is shown in Fig. 8 as function of P and T.

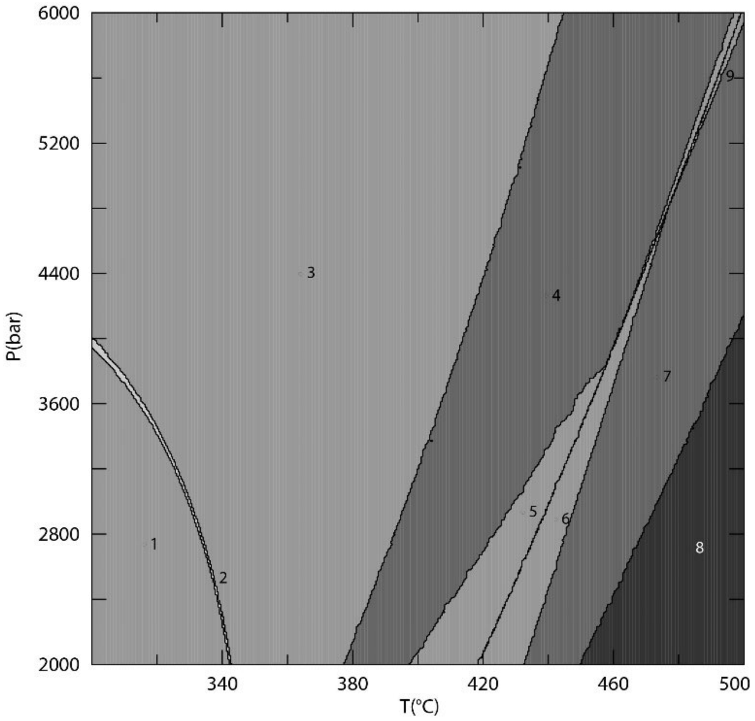


1 - Chl Phe Pl Ep Carb sph ab q H2O CH4
2 - Bio Chl Phe Pl Ep Carb sph ab q H2O CH4
3 - Bio Chl Phe Ep Carb sph ab q H2O CH4
4 - Bio Phe Ep Carb sph ab q H2O CH4
5 - Bio Phe Pl Ep Carb sph ab q H2O CH4
6 - Bio Pl Kfsp Ep Carb sph ab q H2O CH4
7 - Bio Pl Kfsp Ep Carb sph ab q H2O
8 - Bio Pl Kfsp Carb sph ab q H2O

**Fig. S1b** *Perple_X* raw-pseudosection for sample 18 (Mather, 1970), computed with Bio(D). Bulk composition is given in supplementary Table 5. $H_2O$ content was 0.7 wt.%, $CO_2$ and $O_2$ contents were assumed to be 0.3 and 0.015 wt.%.
$K_D = (Fe/Mg)_{Chl}/(Fe/Mg)_{Bio} = 0.88$ in this sample.

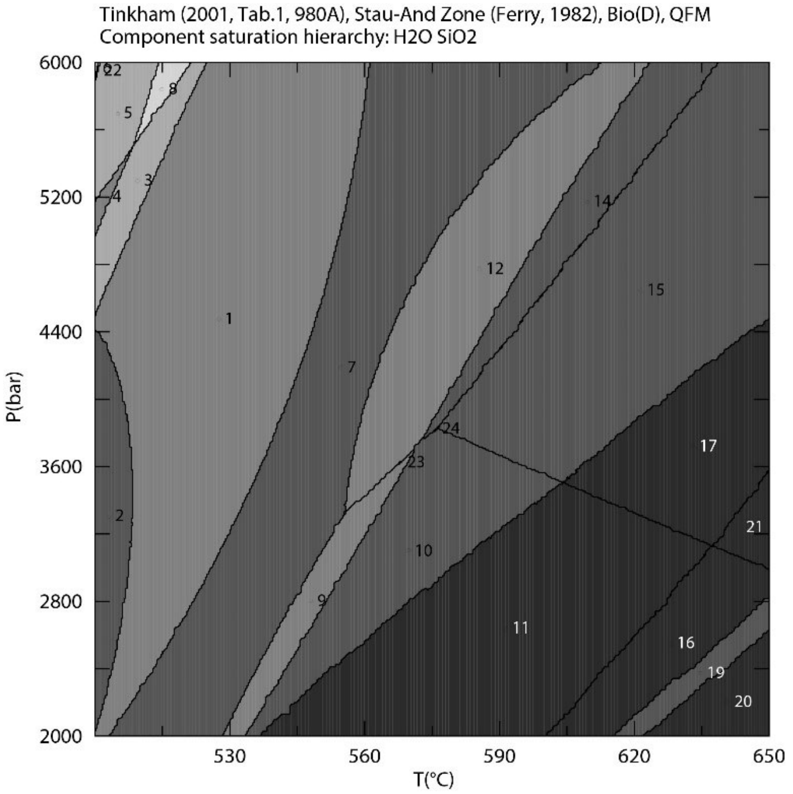


1 - Bio Grt Chl Phe Ilm Pl
2 - Bio Chl Phe Ilm Pl
3 - Bio Grt Chl Phe Ilm Pl ru
4 - Bio Grt Chl Phe Pl ru
5 - Bio Grt Chl Phe Ep Pl ru
6 - Bio Grt Chl Phe Ep Pl sph
7 - Bio Grt Phe Ilm Pl
8 - Bio Grt Chl Phe Ep Ilm Pl ru
9 - Bio Grt Phe Ilm Pl and
10 - Bio Grt Phe Pl and
11 - Bio Phe Pl and
12 - Bio Grt St Phe Ilm Pl
13 - Bio Grt St Phe Pl sil
14 - Bio Grt St Phe Pl
15 - Bio Grt Phe Pl sil
16 - Bio Pl Kfsp and
17 - Bio Phe Pl sil
18 - Bio Phe Pl Kfsp and
19 - Bio Crd Pl Kfsp and
20 - Bio Crd Pl Kfsp
21 - Bio Pl Kfsp sil

**Fig. S2a** *Perple_X* raw-pseudosection for sample 980A (Tinkham 2001, Tab. 1), computed with Bio(D) and imposing logf$_{O2}$-conditions of the QFM buffer. Mineral-chemical data can be found in Ferry (1980). Bulk composition used is given in supplementary Table 5.

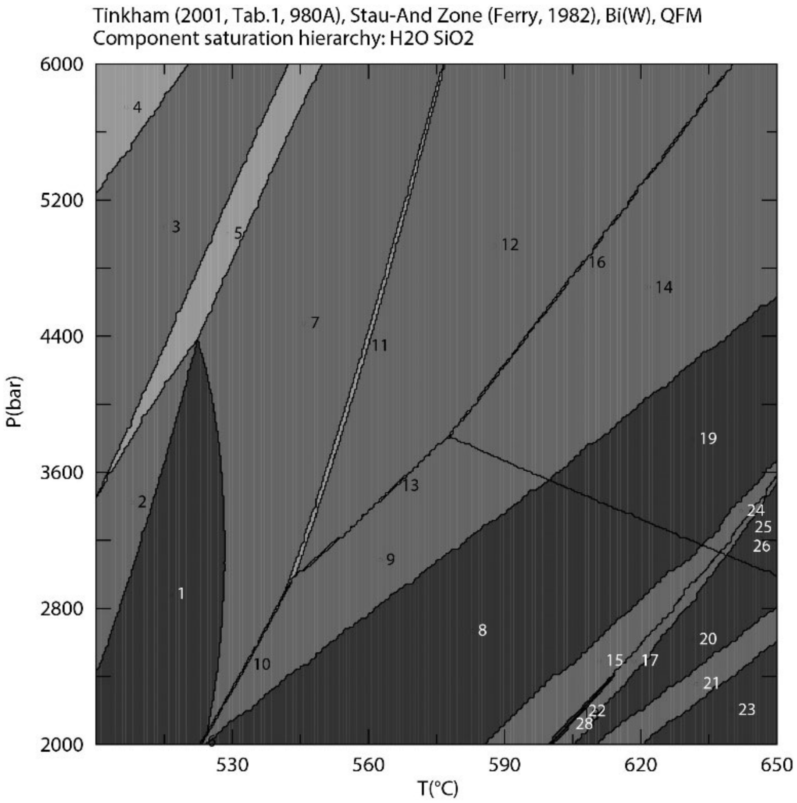


1 - Bio Chl Phe Ilm Pl
2 - Bio Chl Phe Ilm Pl ru
3 - Bio Grt Chl Phe Pl ru
4 - Bio Grt Chl Phe Ep Pl ru
5 - Bio Grt Chl Phe Ilm Pl ru
6 - Bio Chl Phe Ilm Pl and
7 - Bio Grt Chl Phe Ilm Pl
8 - Bio Phe Ilm Pl and
9 - Bio Grt Phe Ilm Pl and
10 - Bio Grt Chl Phe Ilm Pl and
11 - Bio Grt Chl St Phe Ilm Pl
12 - Bio Grt St Phe Ilm Pl
13 - Bio Grt St Phe Ilm Pl and
14 - Bio Grt Phe Ilm Pl sil
15 - Bio Phe Ilm Pl and ru
16 - Bio Grt St Phe Ilm Pl sil
17 - Bio Ilm Pl Kfsp and ru
18 - Bio Phe Pl and ru
19 - Bio Phe Ilm Pl sil
20 - Bio Ilm Pl Kfsp and
21 - Bio Crd Ilm Pl Kfsp and
22 - Bio Pl Kfspand ru
23 - Bio Crd Ilm Pl Kfsp
24 - Bio Phe Ilm Pl sil ru
25 - Bio Ilm Pl Kfsp sil ru
26 - Bio Ilm Pl Kfsp sil
27 - Bio Phe Ilm Pl Kfsp and ru
28 - Bio Phe Pl Kfsp and ru

**Fig. S2b** *Perple_X* raw-pseudosection for sample 980A (Tinkham 2001, Tab. 1), computed with Bi(W) and imposing logf$_{O2}$-conditions of the QFM buffer.

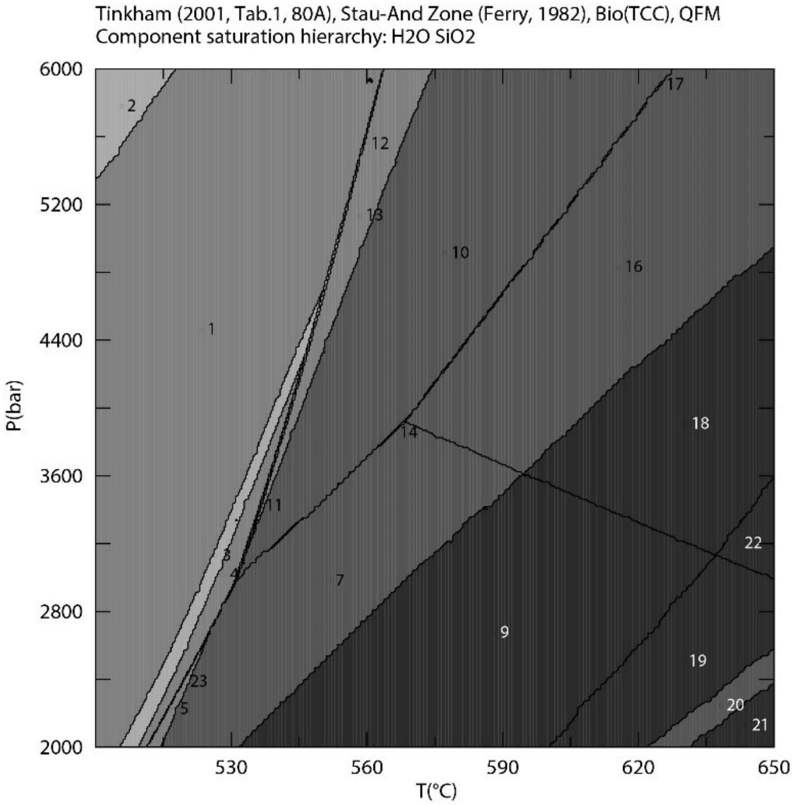


1 - Bio Grt Chl Phe Pl ru
2 - Bio Grt Chl Phe Ep Pl ru
3 - Bio Grt Chl Phe Ilm Pl ru
4 - Bio Grt Chl Phe Ilm Pl
5 - Bio Grt Phe Pl and ru
6 - Bio Grt Chl Phe Ilm Pl and ru
7 - Bio Grt Phe Pl and
8 - Bio Grt Chl Phe Pl and
9 - Bio Phe Pl and
10 - Bio Grt St Phe Pl
11 - Bio Grt Chl St Phe Ilm Pl
12 - Bio Grt Chl St Phe Pl ru
13 - Bio Grt St Phe Pl ru
14 - Bio Grt St Phe Pl and
15 - Bio Grt Phe Phe Pl ru
16 - Bio Grt Phe Pl sill
17 - Bio Grt St Phe Pl sill
18 - Bio Phe Pl sill
19 - Bio Pl Kfsp and
20 - Bio Crd Pl Kfsp and
21 - Bio Crd Pl Kfsp
22 - Bio Pl Kfsp sill

**Fig. S2c** *Perple_X* raw-pseudosection for sample 980A (Tinkham 2001, Tab. 1), computed with Bio(TCC) and imposing log$f_{O2}$-conditions of the QFM buffer.

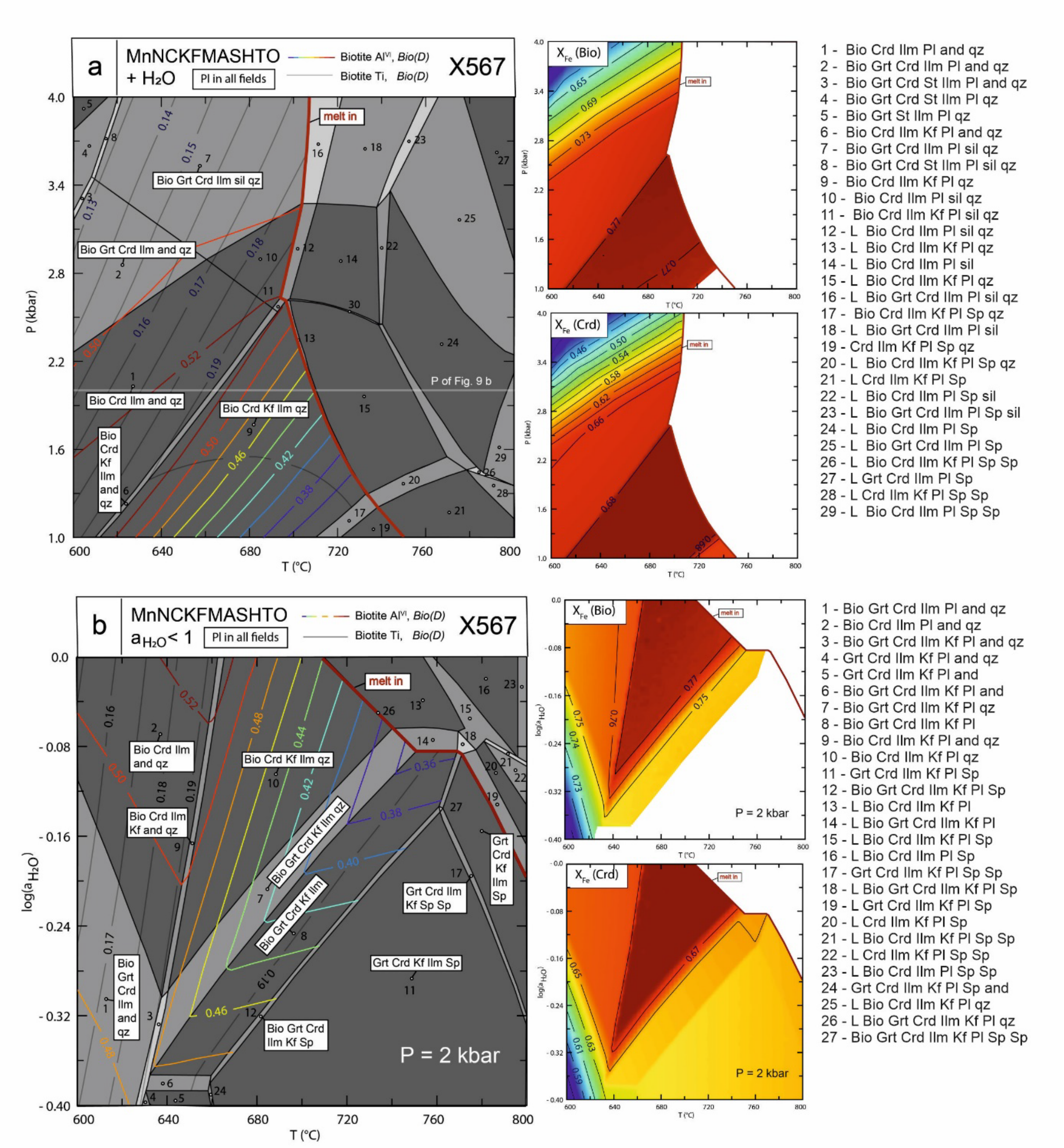


**Fig. S3 a)** P-T pseudosection for water-saturated conditions, **b)** log($a_{H2O}$)-T pseudosection for reduced water-activity conditions at 2 kbar for sample X567 (Pitra and de Waal 2001; bulk-rock composition is given in supplementary Table 5). Calculations were done with *Perple_X* using Bio(D) as activity model for biotite and imposing log$f_{O2}$-conditions of the QFM buffer. Coloured/dark-grey isopleths are the $Al^{VI}$-, respectively the Ti content in biotite, $X_{Fe}$ in biotite and in cordierite are shown in separate colour density plots. Phase-fields are numbered using the following mineral abbreviations: and andalusite, Bio biotite, Crd cordierite, Grt garnet, Ilm ilmenite, Pl plagioclase, Kfsp kalifeldspar, L melt, sil sillimanite, Sp spinel.

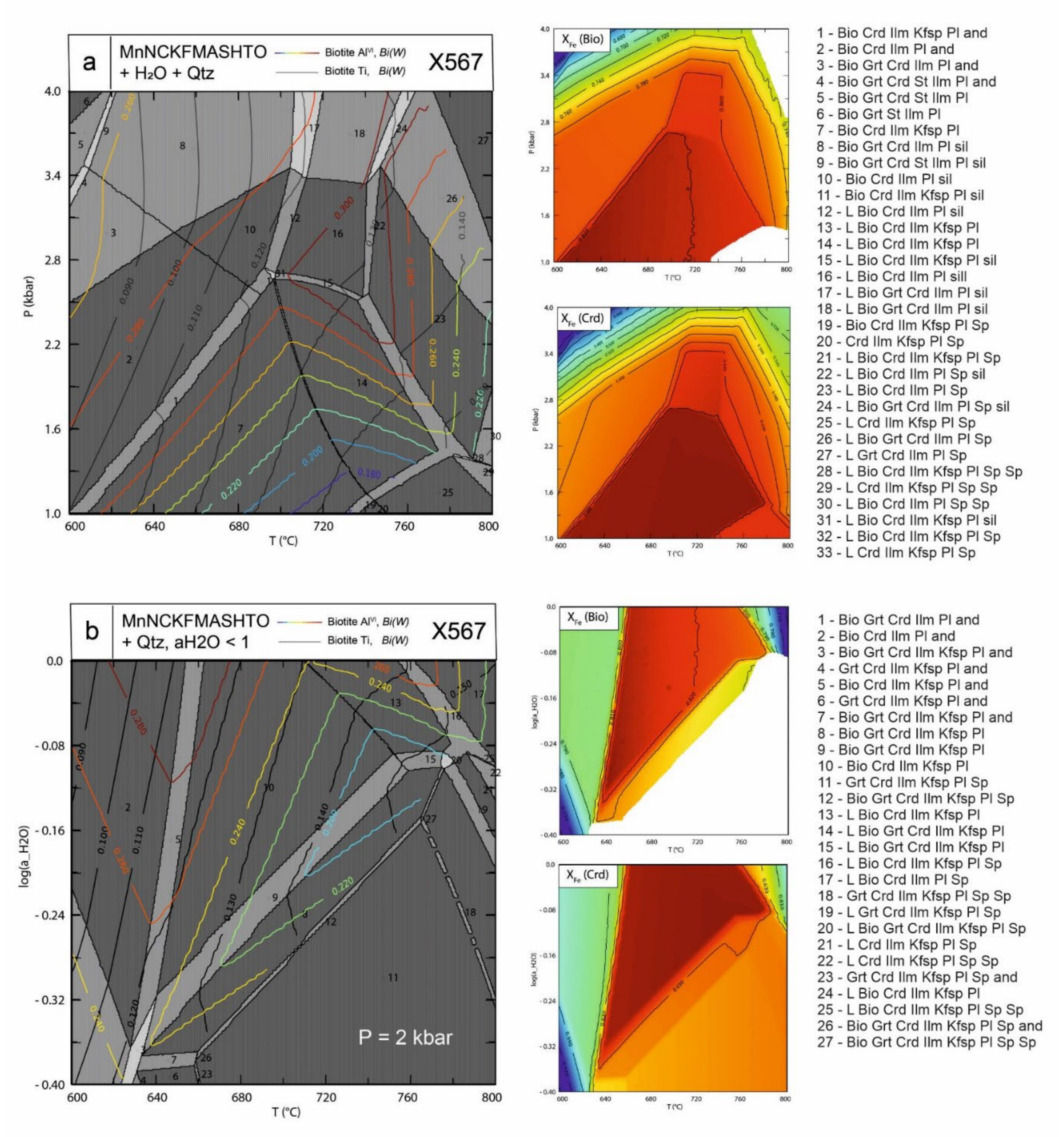


**Fig. S4 b)** P-T pseudosection for water-saturated conditions, **b)** log($a_{H2O}$)-T pseudosection for reduced water-activity conditions at 2 kbar for sample X567 (Pitra and de Waal, 2001; bulk-rock composition is given in supplementary Table 5). Calculations were done with *Perple_X* using Bi(W) as activity model for biotite and imposing log$f_{O2}$-conditions of the QFM buffer. Coloured/dark-grey isopleths are the $Al^{VI}$-, respectively the Ti content in biotite, $X_{Fe}$ in biotite and in cordierite are shown in separate colour density plots. Phase-fields are numbered using the following mineral abbreviations: and andalusite, Bio biotite, Crd cordierite, Grt garnet, Ilm ilmenite, Pl plagioclase, Kfsp kalifeldspar, L melt, sil sillimanite, Sp spinel.

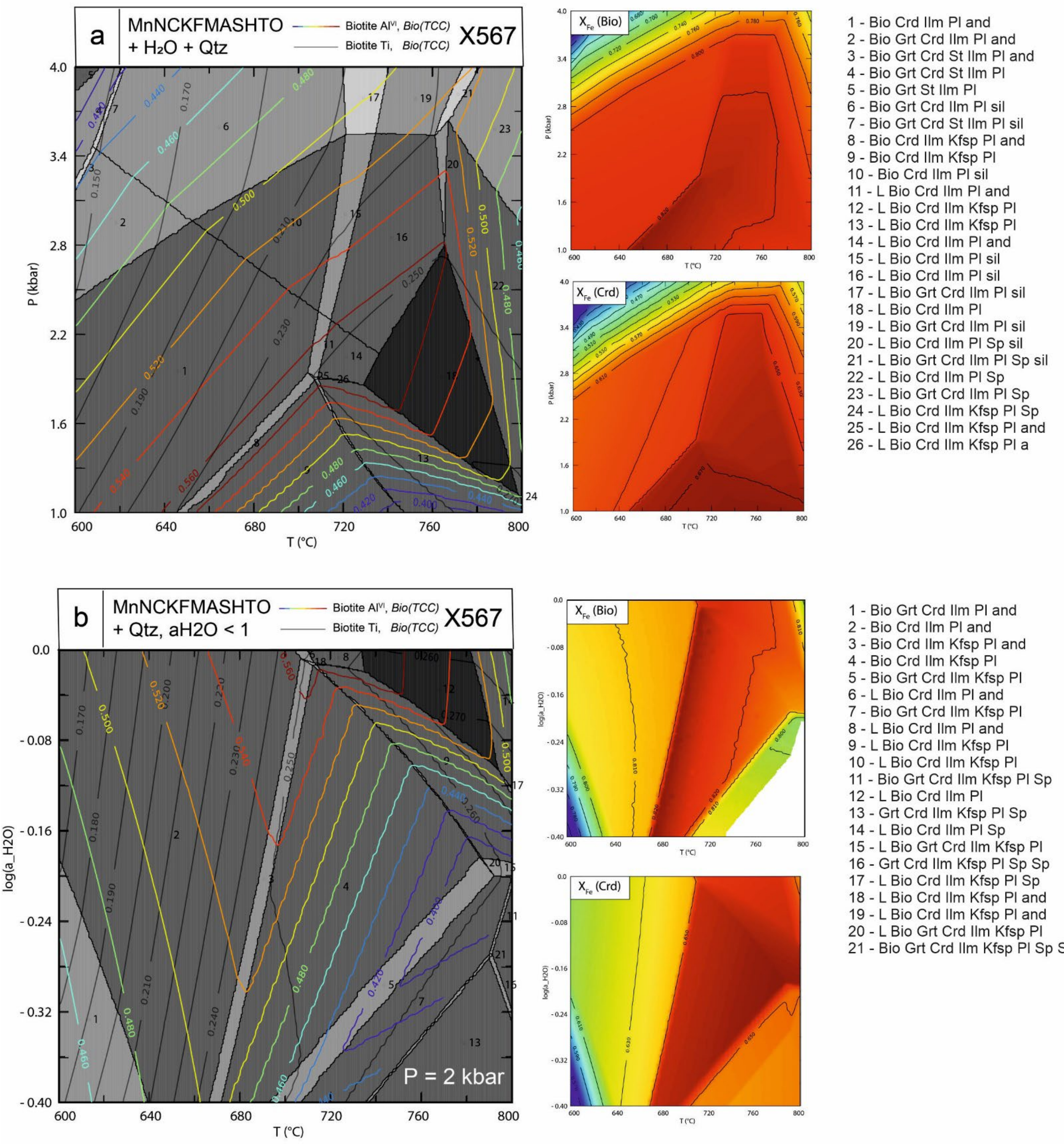


**Fig. S5: a)** P-T pseudosection for water-saturated conditions, **b)** log($a_{H2O}$)-T pseudosection for reduced water-activity conditions at 2 kbar for sample X567 (Pitra and de Waal, 2001; bulk-rock composition is given in supplementary Table 5). Calculations were done with *Perple_X* using Bio(TCC) as activity model for biotite and imposing log$f_{O2}$-conditions of the QFM buffer. Coloured/dark-grey isopleths are the $Al^{VI}$-, respectively the Ti content in biotite, $X_{Fe}$ in biotite and in cordierite are shown in separate colour density plots. Phase-fields are numbered using the following mineral abbreviations: and andalusite, Bio biotite, Crd cordierite, Grt garnet, Ilm ilmenite, Pl plagioclase, Kfsp kalifeldspar, L melt, sil sillimanite, Sp spinel.

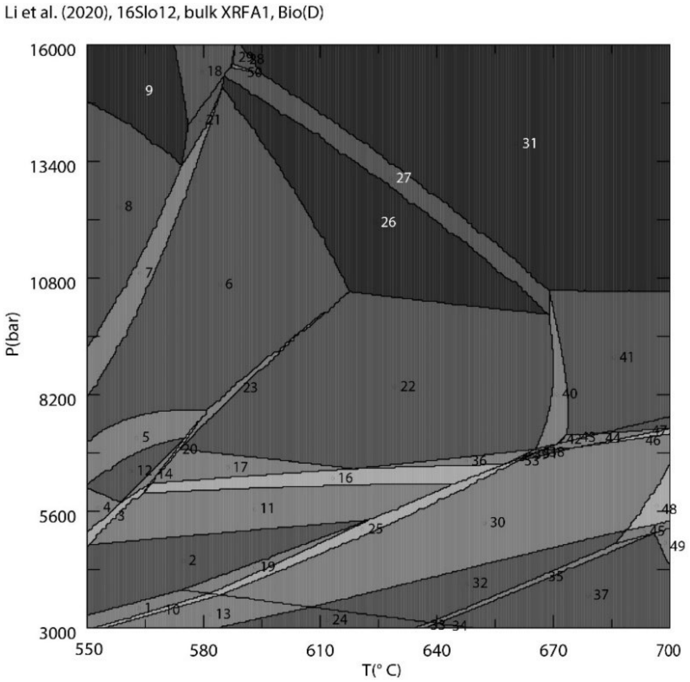


1 - Bio St Phe Ilm Pl and
2 - Bio St Phe Ilm Pl
3 - Bio Grt Chl St Phe Ilm Pl
4 - Grt Chl St Phe Ilm Pl
5 - Grt Chl St Phe Ilm ru
6 - Grt Chl St Phe ru H2O
7 - Grt Chl St Ctd Phe ru
8 - Grt Chl Ctd Phe ru
9 - Grt Ctd Phe ru
10 - Bio Grt St Phe Ilm Pl and
11 - Bio Grt St Phe Ilm Pl
12 - Grt Chl St Phe Ilm
13 - Bio Grt Phe Ilm Pl and
14 - Bio Grt Chl St Phe Ilm
15 - Bio Grt Chl St Phe Ilm Pl ru
16 - Bio Grt St Phe Ilm Pl ru
17 - Bio Grt St Phe Ilm ru
18 - Grt Ctd Phe ky ru
19 - Bio St Phe Ilm Pl sil
20 - Bio Grt Chl St Phe Ilm ru
21 - Grt St Ctd Phe ru
22 - Bio Grt St Phe ru
23 - Bio Grt Chl St Phe ru
24 - Bio Phe Ilm Pl and
25 - Bio Grt St Phe Ilm Pl sil
26 - Grt St Phe ru
27 - Grt St Phe ky ru

28 - Grt Chl Phe ky r
29 - Grt Chl Ctd Phe ky ru
30 - Bio Grt Phe Ilm Pl sil
31 - Grt Phe ky ru
32 - Bio Phe Ilm Pl sil
33 - Bio Phe Ilm Kfsp Pl and
34 - Bio Ilm Kfsp Pl and
35 - Bio Phe Ilm Kfsp Pl sil
36 - Bio Grt St Phe Pl ru
37 - Bio Ilm Kfsp Pl sil
38 - Bio Grt Phe Ilm sil
39 - Bio Grt St Phe Ilm sil
40 - Bio Grt St Phe ky ru
41 - Bio Grt Phe ky ru
42 - Bio Grt St Phe Ilm ky ru
43 - Bio Grt Phe Ilm ky ru
44 - Bio Grt Phe Ilm sil ru
45 - L Bio Phe Ilm Pl sil
46 - Bio Grt Phe Ilm Pl sil ru
47 - Bio Grt Phe sil ru
48 - L Bio Grt Phe Ilm Pl sil
49 - L Bio Ilm Kfsp Pl sil
50 - Grt Chl St Phe ky ru
51 - Bio Grt St Phe sil ru
52 - Bio Grt St Phe Ilm Pl sil ru
53 - Bio Grt St Phe Pl sil ru
54 - Bio Grt St Phe Ilm sil ru

**Fig. S6** *Perple_X* raw-pseudosection for sample 16Slo12 (Li et al 2021), computed with Bio(D). $H_2O$ present in all fields.
